# Open-ended cumulative cultural evolution of Hollywood film crews

**DOI:** 10.1017/ehs.2020.21

**Published:** 2020-05-07

**Authors:** Peeter Tinits, Oleg Sobchuk

**Affiliations:** 1 Faculty of Social Sciences, University of Tartu, Tartu, Estonia; 2 School of Humanities, Tallinn University, Tallinn, Estonia; 3 Max Planck Institute for the Science of Human History, Jena, Germany; 4 Institute of Cultural Research, University of Tartu, Tartu, Estonia

**Keywords:** computational humanities, cultural evolution, films, cumulative culture, innovation

## Abstract

Are there large-scale trends in art history that surpass individual creativity or relatively short artistic movements? Many theories describe art history as a process similar to a change of fashions, while others suggest that art can be progressive – getting better, in some sense, over time. We approach this question anew with the theory of cumulative cultural evolution, which describes cultural accomplishments in terms of innovations that are maintained across generations and accumulated to support ever greater creative potential. In this paper, we empirically test the possibility for cumulative evolution in the techniques used to make an artistic product. Specifically, we measure the size and structure of the production crews in American films in 1910–2010 based on a dataset of 1000 popular films across the century. We find that film crews become exponentially more complex, with a growing set of core jobs, and more innovative in creating new jobs in filmmaking. Our study shows that art history can be cumulative, showing the progressive maintenance of innovative techniques, and thus providing an alternative to the widespread view of art history as a mere fluctuation of trends and fashions.

## Graphical abstract

**Figure:**
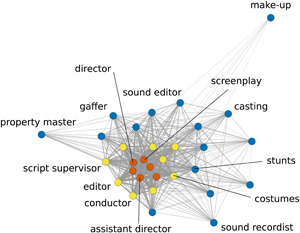

**Media summary:** Is cultural evolution of art cumulative? Historical data analysis of Hollywood film crews shows an accumulation of innovations over 100 years.

## 1. Introduction

Do there exist large-scale principles underlying the evolution of art, encompassing such domains as visual arts, literature, music or film? Past theories, suggesting such principles, can be broadly divided into two kinds. ‘Fashion theories’ claim that artistic genres or styles change in a relatively regular manner: artistic trends come and go, like waves, driven by the preferences of an epoch or sudden creativity of individual geniuses (Fowler, 1982; Bourdieu, 1984; Bloom, 1997). Alternatively, ‘progress theories’ claim that art evolution, at least in some respects, is progressive: artistic techniques are governed by long-term trends and an accumulation of good artistic practices, which allows art, in some sense, to improve over time (Gilbert, 1920; Munro, 1960). While the fashion-like trends have been shown in a number of large-scale studies, exhibiting regular patterns of birth and death of artistic works and genres (Bentley, Lipo, Herzog, & Hahn, 2007; Klimek, Kreuzbauer, & Thurner, 2019; Candia, Jara-Figueroa, Rodriguez-Sickert, Barabási, & Hidalgo, 2018), there has been very little empirical support for progress theories. In this paper we ask whether certain aspects of art – in particular, one type of art, film – demonstrate signs of ‘progress’ similar to the well-documented progress in the history of science and technology (Mokyr, 1990; Pinker, 2018).

*Cumulative cultural evolution* is a concept suggested within the emerging framework of cultural evolution (Mesoudi, 2017; Henrich, 2016), which can prove useful for approaching the progressive theories of art history. According to the idea of cumulative cultural evolution, human cultures are able to accumulate useful innovations from past generations through social learning, which leads to increasingly complex and potent cultural traditions – something that non-human animals seem unable to do (Tomasello, 1999; Dean, Vale, Laland, Flynn, & Kendal, 2014). With the concept of cumulative culture, we could measure ‘progress’ in terms of increasing complexity and potency that relies on inventions of past generations. ‘Generations’ here need not necessarily refer to biological generations, but to generations of cultural practices or items: new versions of technological artefacts (e.g. new generations of iPhones) may equally be understood in evolutionary terms. Recent studies have found cumulative cultural evolution in a number of cultural domains (e.g. in technology (Boyd, Richerson, Henrich, & Lupp, 2013), in programming languages (Valverde, 2016), or in cooking (Lindenfors, Envall, Isaksson, & Enquist, 2015)); however this has not yet been shown for arts.

When talking about domains like art, we consider one aspect of cumulative culture particularly important: its potential for *open-endedness*. Certain types of cumulative cultural evolution imply a development towards greater complexity and creativity as the cultural traits are recombined and reused to create novel combinations, potentially demonstrating exponential growth (Arthur, 2009; Enquist, Ghirlanda, Jarrick, & Wachtmeister, 2008; Kolodny, Creanza, & Feldman, 2015; Winters, 2019). This is not emphasised by all researchers studying cumulative culture: for example, Mesoudi and Thornton (2018) do not include open-endedness in their ‘core criteria’ for cumulative culture, but only among their ‘extended criteria’. At the same time, when we consider the accomplishments of human culture in coming up with creative and effective solutions to their problems, open-endedness seems one of the key characteristics that make humans such unusual animals. Non-human animals can improve their tools and techniques through multiple generations too, but at best this results in what may be called ‘cumulative optimization’ (Winters, 2019): a population is gradually approaching an optimal solution of a particular problem. Open-ended cumulative culture, on the other hand, evolves instead so that the space of possible innovations is progressively being expanded as ‘adjacent possible’ opportunities are explored: novel innovations lead to new opportunities for innovation as the available information can be recombined and seen from a new perspective (Kauffman, 2000; Loreto, Servedio, Strogatz, & Tria, 2016).

Does the evolution of art demonstrate characteristics of open-ended cumulative cultural evolution? To answer affirmatively, we would need to find some works of art that satisfy several criteria. First, these works would have to become progressively more complex, potentially even exponentially more complex, over ‘generations’ of art production. Second, there need to be clear indications of similarities between these artistic works, as new works rely on inventions of the old. Third, we should be able to show a widening scope for potential innovations as more and more elements are available for reuse and recombination.

The artistic complexity and innovations that we have in mind could be measured in different ways. For example, a recent study investigated the visual structure of almost 140,000 paintings during the last millennium and found the complexity and entropy to systematically vary by artistic era, however with no consistent growth or decrease across the period (Sigaki, Perc, & Ribeiro, 2018). Other researchers, exploring the role of the ‘adjacent possible’ in innovation, traced the sequences in which particular words and tags are used in composing encyclopaedia articles, social media annotations or literary works (Tria, Loreto, Servedio, & Strogatz, 2014; Monechi, Ruiz-Serrano, Tria, & Loreto, 2017). We think that there is a layer of organisation in artistic works that could be relevant for understanding the evolution of art, but may be difficult to capture by only inspecting the formal characteristics of the work, such as the words used in it or its visual structure. Particularly, art historians would say that artistic works usually combine a number of diverse techniques to make their final form (e.g. linear perspective (Kubovy, 1986), the use of photographic examples (Stromp et al., 2018) or violet colour (Tager, 2018) all make up the complexity of the painting). However, finding them may cause problems even for trained art historians, not to mention naive viewers of art works. We propose that there is a way to systematically explore this aspect of artistic works. In particular, the variety of techniques used to produce an artwork can be seen more clearly when looking at the *production process* itself: namely, at the shape and structure of the effort put into making an artwork. For most art forms, this collective effort may be difficult to track, while for the others, such as film, it can be well documented over a long period.

Such an approach may raise a question: what is actually being studied – a process of production or a product, a finished artwork? And what, potentially, becomes more complex: the process or the product? We think that to do justice to the evolutionary nature of culture, we should avoid such a sharp distinction. Instead, following the work of the sociologist Howard S. Becker (2008), we consider art as an activity. The final product – say, a book – is only a small part of the activity of making a book, some of which is, to greater or lesser degree, acknowledged and reflected in the finished product (the work of an author, the editor, sometimes the translator, etc.), while many other essential components of book production as an activity often remain invisible (say, the author's agent, the organisers of book promotional tours, etc.). In other words, the book *is* the activity of making a book. Producing a book is a radical example of how information about a multitude of collaborating people is virtually lost, and the finished product is attributed, in its entirety, to a single person: the author. However, there are artforms where the collective nature of the enterprise is more evident and better documented. Such artforms can be more suitable for studying art as an activity.

In this study, we develop a test for an accumulation of production complexity in one type of art – films. More precisely, we analyze production crews able to make films that are well received by the audience. A production crew includes all of the people involved in making a film, excluding actors. We argue that job titles of the film crew members reflect the tasks performed to complete the film. Whenever new useful tasks are introduced, for example owing to technological advances or novel artistic techniques (e.g. sound editor, assistant director or CGI artist), these jobs tend to be reused in other film production crews – potentially even becoming a standard in filmmaking. The presence of particular jobs in a crew thus indicates the mix of artistic techniques and special skills required to create one artistic product – a film.

As our test case, we take the history of Hollywood films during 1910–2010. We argue that the Hollywood film industry should be particularly prone to cumulative evolution. Film production studios work in a highly competitive environment: they aim to please large numbers of viewers and are under a constant pressure to manage their resources to obtain that goal (de Vany, 2004). In art theory this is known as a ‘heteronomous’, or market-oriented, artistic field, which follows quite different principles from ‘autonomous’ fields, where the evaluation of good artworks largely depends on peers and critics (Bourdieu, 1993). In short, autonomous fields tend to produce ‘art for art's sake’, while heteronomous fields tend to produce ‘to sell’. In a heteronomous field, art producers are extremely interested in using the experience of other successful artworks in their own work, while in an autonomous field, artists are more free to follow their interests. As a result, the heteronomous film market should offer a good example of cumulative cultural evolution, in contrast to, for example, modern painting, which can afford to develop in a fashion-like manner.

To see if some sort of ‘progressive’ development could be seen in the film industry, we measure several characteristics that may be jointly indicative of open-ended cumulative cultural evolution among the popular films. First, we measure the complexity of the film crews through the number of people and jobs involved in producing a film. Then, we track the accumulation and maintenance of innovations in jobs that are preserved from past generations. Finally, we explore the recombination of job components and the exploration of the innovation space and its implications for the creative potential within the cultural system.

We find that film production crews were becoming more complex during the observed period. The size of the film crews shows an exponential increase during 1910–1939 and 1967–2010. The particular jobs that were used show a dynamic of accumulation of core jobs: when any job becomes widely used, there is only a small chance of it falling out of common use in the subsequent decade. The jobs became increasingly reused within and between films, and are organised into hierarchical clusters of specialised jobs over 100 years. Finally, the increased variation between the used jobs supported the growing expansion of the innovation space, as novel jobs came to be invented at an increasingly quicker pace. The cumulative cultural evolution that we find provides support for the progressive theories of art history, at least when it comes to Hollywood film production. More specifically, such phenomena as growth of complexity, maintenance of innovations and growth of innovation space may apply to art as well as they do to technology.

## 2. Data and methods

### 2.1. Data

To collect information on film crews, we relied on the Internet Movie Database (IMDb; https://www.imdb.com/), a website that aggregates various information on the production and reception of films. It includes information about people involved in the production of each film and their particular roles in that film. The database strives to also include the people who were not credited on the release of the film. At the time when the data was collected (14 April 2019), IMDb included information on 506,296 feature films, a number close to the total of all films ever produced (see Supplementary Information, Section S1 on data collection).

We selected the 100 most popular films for each decade, according to IMDb users’ ratings, during 1910–2010, resulting in a sample of the 1000 most popular films for the period. Popular films are a convenient dataset to study, as they are comparable over time: popular films accomplished the main task of the film industry – to produce a well-received film. By analyzing these films we are analyzing what it took to make a well-received film at the time, at least based on modern ratings. On IMDb, data quality is also expected to be better for popular films, as public interest has probably increased efforts at data collection. In our sample, two-thirds of the films were marked as having their crew data verified as complete or expected to be complete. The degree of confidence in the data was also taken into account in the analysis (see Supplementary Information, Section S2 for details on data reliability).

#### Sample formation

To form the sample of most popular films per decade, we relied on votes from IMDb users, who provided ratings of 1–10 for each film. In order to maintain comparability between films, we limited the sample to non-documentary feature films that were, at least partly, produced in the United States and in English language. Excluding films with fewer than 50 votes, we took 1000 films with most votes per decade and from them took the 100 films with the best average ratings. In case of ties, we preferred films that had their crew information marked ‘verified as complete’ or ‘expected to be complete’ and then a higher number of votes. This resulted in a sample of 1000 films, evenly distributed over 10 decades.

#### Jobs connected to release

We collected the information about the film crews from the IMDb website. We excluded from the dataset all jobs that were linked to a time after the initial release (e.g. special editions, a director's cut, or a musical score added several decades after) or were marked as unexpectedly short (e.g. when a person was indicated as ‘fired’). When job titles listed several roles for a person in one entry (e.g. ‘helicopter pilot and/or camera operator’), word processing heuristics were used to split this into separate jobs performed by the same person (see Supplementary Information, Section S3). After these transformations, the data amounted to 147,808 job entries.

#### Harmonisation of job titles

To measure job reuse and complexity, we removed the additional specifics that were sometimes included in job names (e.g. ‘stand-in: Humphrey Bogart’, ‘animal wrangler: birds’). We also removed information on whether they were included in film credits or whether they were given a different alias in them (see Supplementary Information, Section S3).

### 2.2. Measurements

#### Crew complexity

We measured the number of individuals involved in production of a film, the number of jobs allocated to them and the number of unique jobs in each film. These measures were highly intertwined and thus also highly correlated (*R*^2^ > 0.95 across all pairs; see Supplementary Information, Section S5).

#### Job title length

We counted the number of words in each job title and computed the mean length of job titles per film. This was done after the job titles were harmonised.

#### Hierarchical order of jobs

To measure the placement of jobs within hierarchical orders, we checked the presence of specifiers that could be associated with superordinate (e.g. ‘chief’, ‘boss’, ‘key’, ‘1st’) and subordinate jobs (e.g. ‘assistant’, ‘additional’, ‘2nd’), as well as specifiers that bore a neutral association mark (e.g. ‘collaborating’, ‘associate’, ‘advisor’, ‘consultant’). Each job could bear one or several markers of hierarchical structure (see Supplementary Information, Section S4).

#### Reuse of elements

We measured the reuse of elements through the type-token ratio (i.e. the number of unique elements divided by the number of total elements), for the job titles and for job title components. This was done after harmonisation. Subtracting this ratio from 1, we got the proportion of repetitions within the set.

#### Innovation space

For each unique job title, we allocated a position in a unidimensional innovation space based on their order of appearance. This was done separately for each thematic job cluster based on the words within the job title.

### 2.3. Data analysis

We analysed the trends over time for the three measurements on crew complexity, job title length, the markers of hierarchical order, job reuse and job component reuse with a generalised additive model (GAM), with the following formula:
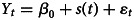
where *Y* is the measured response at year *t*, *s*(*t*) is the smooth function of time, *β*_0_ is the intercept and *ɛ_t_* is the residual error. A generalised model allows the shape of the fitted trend to be based on the data and processes that discourage both over- and underfitting to the data (Ruppert, Wand, & Carroll, 2003; Wood, 2017). We modelled the crew size metrics on a logarithmic scale with a Gaussian error distribution. The proportion of jobs with hierarchy markers and the proportion of repetitions of jobs and job components were modelled with a beta distribution, truncated at 0 and 1. The mean job title length was modelled on a linear scale with a Gaussian distribution. All models were estimated with the restricted maximum likelihood estimator with 15 basis dimensions. In order to allow for sudden changes and periods of relative stability and change, an adaptive smooth regression spline with five smoothing parameters was used. An adaptive smooth allows the wiggliness of the smooth to vary over the observed period. See details in the Supplementary Information, Section S6.

The periods of significant change were identified based on the first derivative of the fitted trend. Derivatives of the fitted spline were estimated using the method of finite differences. The periods of significant change are the time periods where the Bayesian credible interval on the first derivative does not include zero (Simpson, 2018). These intervals were obtained by simulation from the posterior distribution of the first derivative. A 95% credible interval here contains in its entirety 95% of all random draws from the posterior distribution (Simpson, 2018). This is also known as a simultaneous interval (Wood, 2017). GAMs were estimated using the *mgcv* package, version 1.8-28 (Wood, 2017), for R, version 3.6.0.

For the analysis of the expansions of innovation space in relation to variety of jobs present, we constructed two linear regression models – (a) for the sum of all jobs and (b) for the expansion of innovation space within thematic clusters – allowing for a random intercept and slope for each theme. In both cases the model selection and criticism led us to include the tempo of growth as a predictor to establish a good fit to the data. The model formulas were as follows:1
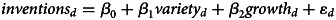
Here, *inventions_d_* is the log-transformed number of jobs invented in decade *d*, *variety_d_* is the log-transformed number of jobs reused from the past decades in decade *d*, *growth_d_* is the proportional increase in the number of total jobs compared with the prior decade for decade *d*, *β*_1_ and *β*_2_ are the fixed effects slopes, *β*_0_ is the intercept, and *ɛ*_*d*_ is the residual error.2
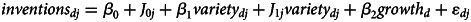
Here, *inventions_dj_* is the log-transformed number of jobs invented in decade *d* for job theme *j*, *variety_dj_* is the log-transformed number of jobs reused from the past decades in decade *d* for job theme *j*, *growth_d_* is the proportional increase in the number of total jobs compared with the prior decade for decade *d*, *β*_1_ and *β*_2_ are the fixed effects slopes, *J*_1*j*_ is the random slope for job theme *j*, *β*_0_ is the intercept, *J*_0*j*_ is the random intercept for job theme *j*, and *ɛ_dj_* is the residual error.

## 3. Results

### 3.1. Growing complexity

The complexity of the film production crew as a cultural system can be measured through its number of parts. This is similar to how technological complexity has been measured in terms of the techno-units that it consists of, i.e. distinct configurations that make up the artefact (Oswalt, 1976). For film crews, we can find close analogues to techno-units in the jobs that make up a film crew and the people who perform these jobs. We thus measure film crew complexity through three parameters to capture the number of techno-units: the number of people associated with each film, the number of roles they were given and the number of unique job titles used within a film.

**Figure 1.**
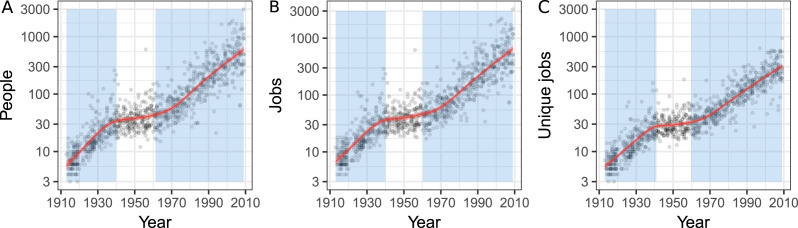
Sizes of film crews in 1910–2010 (*n* = 1000). (a) Number of people; (b) number of jobs; and (c) number of unique jobs per film. The *y-*axis is on logarithmic scale. The red line depicts the predicted means of the GAM with a 95% confidence band around it in pink. Blue areas mark the periods where, at a 95% credible interval, the increase significantly differed from 0. Significant decrease was not observed.

We calculated these measures for each film and found that over the observed period each of the measures shows a pronounced increase (see Figure 1). From the 1910s to the 2000s the mean number of people involved in a film grew from 8.0 to 604.1, and the mean number of jobs associated with the film – from 9.2 to 655.3, and the mean number of unique jobs – from 7.2 to 283.4. For example, a hit movie early in film history, *Frankenstein* (1931), had a crew composed of only 45 people. A hit movie of the 2000s, *The Dark Knight* (2008), had a crew size of 1438 people.

The growth was not evenly spread, so we fitted a GAM to each of the measures to investigate the nonlinearities in the growth patterns (here, we used the balanced models that weighted the data points according to their known completeness; see more in Supplementary Information, Section S6). We found periods of significant growth around 1913–1940 and 1960–2010 for each measure. During these periods the growth was exponential: the number of people increased by 5.9% per year, the number of jobs by 5.8% per year, and the number of unique jobs by 5.0% per year. This agrees with our understanding that cumulative cultural evolution should lead to an increase in cultural complexity, in this particular case even following an exponential pattern of growth. However, in order to support the case for ‘progress’ in arts, we would have to show that there is also a clear accumulation of innovations from past generations and an increase in creative potential among the film makers.

### 3.2. Innovations in jobs accumulate

Cumulative cultural evolution entails that newer generations build on the innovations of past generations. This phenomenon takes the form of a ‘ratchet effect’ with the maintained innovations allowing new problems to be tackled (Tomasello, 1999; Tomasello, Kruger, & Ratner, 1993). For the film crews this would mean that innovative jobs would increase in popularity gradually and would remain popular once they had become so. As a result, over time a core set of jobs would emerge that would be used in many films, and their number would increase over time.

**Figure 2.**
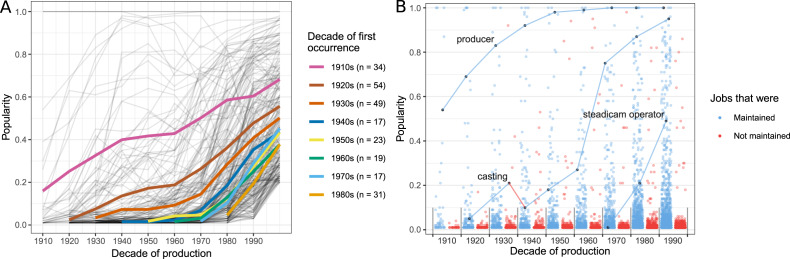
The gradual diffusion of jobs. (a) Diffusion curves of jobs that were in at least 20% of films in the 2000s and originated before the 1990s (*n* = 244). Coloured lines mark averages by decade of origin, *n* shows the number of jobs on the plot that originated from these decades. Grey lines show the trajectories of individual jobs (see Supplementary Information, Section S9 for each decade separately). (b) The maintenance of jobs in each decade by their prevalence. The dots mark the relative popularity of a job in that decade. Blue dots mark jobs that proved stable into the next decade (*n* = 4747); red dots jobs that proved unstable (*n* = 5565) following the set threshold. The lines indicate diffusion trajectories of three selected jobs: ‘producer’, ‘casting’ and ‘steadicam operator’.

We can measure the popularity of a job by calculating the fraction of films that contain the job. We tracked the diffusion of jobs that ended up being fairly popular in the 2000s: jobs that were in at least 20% of the films (Figure 2a). Apart from the jobs that originate in the first decade, the 1910s, most of the jobs were rather uncommon in the decade after their first occurrence in the sample. Accordingly, except for a few basic jobs that were introduced at the very beginning (e.g. ‘director’), the diffusion of jobs was gradual. At the same time, the results show that each decade offered a considerable number of innovations that eventually became commonly used (mean, *M* = 30.5; standard deviation, *SD* = 14.4).

We also studied the mechanism responsible for this gradual diffusion by comparing the popularity of a job in our sample between two consecutive decades. The ‘ratchet effect’ would predict that jobs that had become commonly used would remain so throughout the time period. We tested this by comparing the relative popularity of jobs between consecutive decades. A job was considered maintained if it had similar or higher popularity in the next decade. To account for possible random fluctuations, we considered the level of popularity as similar even if the job was slightly less popular (we allowed for fluctuations up to 10% of the sample, i.e. 10 films).

The results are plotted in Figure 2b. We found that across the decades around half of the jobs could be considered maintained (*M* = 48.2%, *SD* = 6.4%, *n =* 10,312). However, when the jobs were currently popular, they were much more likely to be maintained. The jobs that were in less than 10% of films in a decade showed a slightly lower turnover than the total population (*M* = 43.2%, *SD* = 6.0%, *n* = 9419). At the same time, the jobs that were in 10% of films or more had a very high chance of being maintained across decades (*M* = 91.2%, *SD* = 5.5%, *n* = 893). Thus, the jobs that were already popular had a much higher likelihood of remaining popular in the next decade.

**Figure 3.**
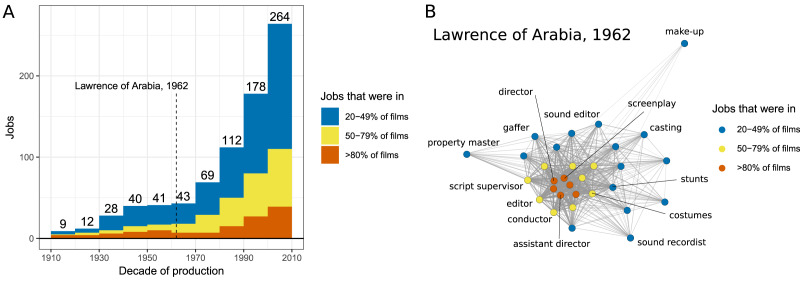
The accumulation of popular jobs. (a) The number of jobs shared by 20% or more films in each decade. Colour indicates popularity in that decade: central jobs (in >80% of films) in blue, moderately popular jobs (in 50–79% of films) in yellow and somewhat popular jobs (in 20–49% of films) in red. The numbers above the bars cumulatively count all jobs in more than 20% of films. (b) A network representation of one film as an example. The nodes are coloured by their popularity in that decade to match (a). Network links are weighted by the proportion of occurrences in which the jobs appeared together. Jobs in less than 20% of films are excluded from both plots. For illustration, we labelled selected nodes with their job titles.

Finally, we looked at how many jobs were commonly used in each decade. Figure 3a shows the accumulation of jobs that were in at least 80, 50 and 20% of the films overlaid on top of each other. The number of jobs in each category grew a lot over the 100-year period (e.g. the 1910s had five jobs in at least 50% of the films; the 2000s had 110 jobs in the same category). Based on their prevalence, we can understand the jobs used in films as relatively central or relatively peripheral. Central jobs are present in most films and make up the core of the crew organisation, while peripheral jobs are present in just a few films and can be seen as less important for filmmaking. In order to illustrate this idea, we present the job network for one film, *Lawrence of Arabia* (1962), based on the proportion of cooccurrences of jobs within films in the 1960s (Figure 3b).

In order to check that the growth of central jobs is not simply due to growth in the sizes of film crews (in this case they could share more jobs simply by chance), we created 1000 random datasets with the same set of jobs (see Supplementary Information, Section S8 for details). The generated data showed that, owing to a growth in unique jobs that accompanied the growth in crew sizes, the number of jobs shared between many films stayed roughly the same throughout the period, which, except for the first decade, is decisively lower than in our sample (*M*_MEAN_ = 12.7, *SE*_MEAN_ = 6.1, *M*_SD_ =3.0, *SE*_SD_ = 0.7 between decades). It was highly unlikely that any job was shared by more than 20% of the films by chance. This indicates that the growth in the number of jobs shared between films is indeed due to an accumulation of innovations that become preferentially used between films.

In sum, our data indicates that, over the century, innovations in jobs accumulated and were actively maintained by the producers of popular films. Over time, an increasing number of jobs became central to film production process, forming a stable core. The increase in the complexity of the film crews thus manifests not only in their relative growth, but also in a growing maintenance of innovations in jobs that probably allow the film industry to better achieve its goals.

### 3.3. Increased recombination and the growth of innovation space

According to the definition of open-ended cumulative culture, we must expect active recombination of job components, possibly leading to the emergence of more specialised jobs, as well as the increased reuse of job components. For film crews, we trace this through two types of data: explicit markers of specialisation and hierarchical order of the jobs; and the reuse of jobs within a film crew. In both cases, we would expect to see trends of increase. Additionally, the accumulation of popular jobs, discussed in the previous section, and the expected higher rate of recombination of job components should lead to the growth of the number of possible combinations: the ‘innovation space’ ought to increase as the variety of elements available for reuse increases.

Frequent reuse of jobs within the film crews would naturally make them more specialised and often place them into a hierarchical order with regards to other jobs, forming sets of jobs that function well together.

**Figure 4.**
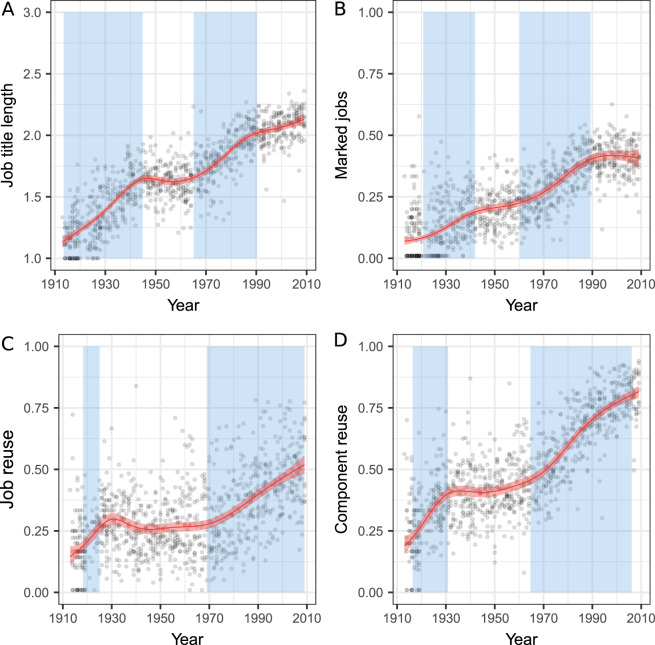
Film crew structure in 1910–2010 (*n* = 1000). (a) Mean length of job titles; (b) proportion of jobs with the markers of hierarchical structure; (c) job reuse ratio; and (d) job component reuse ratio. Red line depicts the predicted means of the GAM with a 95% confidence band around it in pink. The blue areas mark periods where, at a 95% credible interval, the increase significantly differed from 0. Significant decrease was not observed.

For a proxy of specialisation, we measured the number of words in a job title: a title with more words is expected to correspond to more specialised tasks (e.g. ‘editor’, 1; ‘assistant director’, 2; or ‘special effects assistant’, 3). From the 1910s to the 2000s, mean job title length increased from 1.2 to 2.1 words per job per film. We fitted a GAM to check for nonlinearities in the trend (Figure 4a) and found, similarly to other parameters, a period of no detectable growth in 1945–1965, with another slowdown from 1990 onwards. While the 1945–1965 period may be subject to similar constraints to other parameters, the slower growth at the end of the period may indicate a different dynamic: possibly, the titles had become long enough to allow various specialisations.

To measure the hierarchical order of jobs, we took a qualitative look at the job titles. Many jobs contained an explicit marker of their position in a hierarchy (‘*supervising* editor’, ‘*assistant* director’, etc.). We assembled a small vocabulary of such keywords marking hierarchy (see Supplementary Information, Section S3 for details) and tracked the presence of such markers among the job titles in each film. We found that the proportion of jobs with hierarchy marking steadily increased from an average of 10.7% in the 1910s to 40.8% in the 2000s. The GAM analysis showed a significant growth from 1920 to 1942 and from 1960 to 1989 (Figure 4b). Considering the types of markers separately, we found that subordinate jobs showed a pattern of growth similar to the increase in film crew size with a period of stability during 1940–1960, while superordinate jobs grew gradually throughout the century (see Supplementary Information, Section S7).

We measured the reuse of elements within the system by calculating the type-token ratio (i.e. the number of unique elements divided by the number of total elements) for each film for job titles (Figure 4c) and for the words within job titles as their components (Figure 4d). This gave us the proportion of first occurrences, and when we subtracted it from 1, we obtained the proportion of elements that were repetitions of another element. The reuse of job titles grew from 19.1% of repeated jobs in the 1910s to 49.0% in the 2000s. The reuse of the job title components grew from 24.8% of repetitions in the 1910s to 80.1% in the 2000s. This growth also took place in the beginning of the period – 1918–1924 for job titles and 1916–1930 for job title components – and then later during the time of growth in film crew size – from 1968 or 1965 until near the end of the period, respectively.

If cumulative culture results in an accumulation of openness and creativity in the system as an increasing variety of elements are available for reuse, we should also see this in the data. That is, as the variety of elements increases, we should see more innovations and novel combinations to be made in the system. As a result, the growth in complexity will have led to increased possibilities for future development in the system.

**Figure 5.**
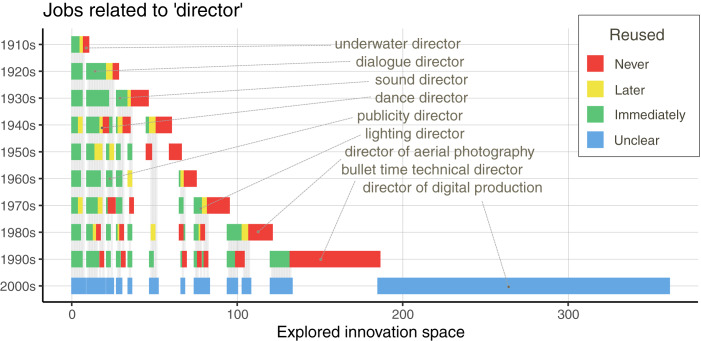
The discovery of jobs in the thematic cluster of ‘director’ in the top 100 films. The graph shows on the *y-*axis the decade and on the *x-*axis the jobs that were in use that decade (*n* = 567 unique job–decade combinations). Each unique job is given a stable location (*n* = 360) across decades, sorted by their decade of origin and their reuse in later decades. The grey lines connect the instances of the same job across decades. The colour indicates whether the job was reused immediately in the next decade (green), at a later decade (yellow), never again (red), or unclear owing to the lack of data on the decade after (blue). The right edge of the coloured line indicates the space of possible innovations explored by the end of the decade.

It is difficult to study what may have been, but it is possible to track how the space of possibilities came to be explored. For example, there are 360 job titles that are a variant of ‘director’, i.e. contain the word ‘director’. It seems reasonable to suggest that these jobs are related to each other in some sense, forming a natural cluster. Tracing the way these jobs came to be invented can thus give some indication as to the creative potency within the system; each new invention is combining the ‘director’ component with something else. Figure 5 shows how these 360 variants were discovered across decades. Some were forgotten quickly, e.g. ‘underwater director’ in the 1910s, ‘vocal director’ in the 1940s, ‘director of aerial photography’ in the 1980s. Others became part of the core of filmmaking, e.g. ‘art director’ from the 1910s, or ‘additional assistant director’ in the 1980s. What is notable here is that the tempo of discovery speeds up as the number of variants from past decades increases.

**Figure 6.**
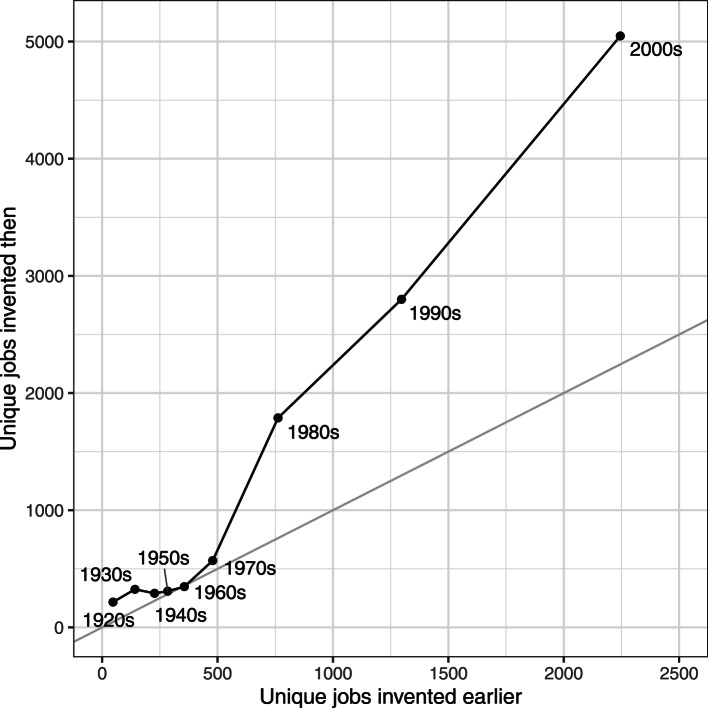
The number of jobs invented in a decade (*y*-axis) vs the number of jobs reused from the previous decades (*x*-axis). Grey line, where *x* = *y*, shows the hypothetical case where the number of inventions in a decade equalled the number of jobs from the previous decades.

When we look at this across all jobs, we see a clear trend: the number of inventions is closely tied to the variety of jobs from previous decades used within the populations (see Figure 6). The log-transformed number of invented jobs shows a high correlation of 0.90 with the number of unique jobs present that originated from earlier decades. This is partly modulated by the growth in the overall population: it is likely that slower growth rates allow fewer innovations. A linear regression model, combining the variety present and the overall growth rate compared with the prior decade, explains 96% of the variation in the data (*β*_variety_ = 0.87 95% CI [0.71–1.04], *β*_growth_ = 0.97 95% CI [0.54–1.39], *F* (2,6) = 103.9, *p* < 0.01, *R*^2^ = 0.96). This relationship also holds across thematic clusters of jobs, whereby the number of inventions within the cluster is highly associated with the variety reused from earlier decades. For this, we took the 352 thematic clusters that had at least 10 related jobs explored in the innovation space (space explored median = 28, interquartile range = 16–56, range = 10–1266), and fitted a mixed effects model with the same parameters adding a random intercept and a random slope for the log-transformed old jobs predictor for each job cluster (see Supplementary Information, Appendix S10 for details), and found the association to be strong (*β*_variety_ = 0.77 95% CI [0.72–0.82], *β*_growth_ = 0.71 95% CI [0.62–0.80], with marginal *R*^2^ = 0.57 and conditional *R*^2^ = 0.70), demonstrating the close link between the variation already present in the population with inventions produced in this area of culture. The strong relationship within thematic clusters shows how the cultural system allowed for more innovation when there were more elements to be developed and recombined within the theme.

## 4. Discussion

By looking at the production crews associated with the release of 1000 popular films during 1910–2010, we have discovered a gradual increase in complexity of film production. This increase can be understood in terms of cumulative cultural evolution: the cultural system grew in complexity, preserved innovations from the past and as a result became more potent in exploring open-ended innovations in the future. This provides support for the progress theories of art: at least in some aspects, we can see a gradual accumulation in art history, which supersedes the preferences of the current epoch.

These findings complement the existing work on collaboration networks in science and art (Guimerà, Uzzi, Spiro, & Nunes Amaral, 2005; Uzzi & Spiro, 2005; Wuchty, Jones, & Uzzi, 2007). These studies have documented how the networks of scientific collaborations have grown significantly in recent history, which is similar to our finding. For the domain of art, a trend of growth in production crews has been found for Broadway musicals (Guimerà et al., 2005) – however, differently from our findings, this growth seems to have peaked mid-century. We see two reasons why films would notably differ from Broadway plays in this trend: (a) Hollywood films belong to the heteronomous artistic field – they are products that need to be sold on a mass scale, and as a result they are subject to strong selective pressures, which can be interpreted by the producers as clear signals on which innovations are effective and which are not; and (b) film as a medium allows for a potentially limitless amount of effort to be placed in every frame, while in musicals the cast and crew that can fit on the stage are likely to be much more strictly bounded. As a result, the crews of the Broadway musicals may have been dealing with ‘cumulative optimization’ (Winters, 2019) to fit the narrow constraints of the stage play instead of open-ended cumulative evolution that films may be able to support.

Apart from the pattern of growth, we have also discovered a slowdown in the growth of most measured parameters in the 1940s–1950s. This may be explained by some external events that considerably shocked the Hollywood film industry. Apart from the global influence of the Second World War, the 1940s also initiated the spread of television, which quickly led to a threefold drop in cinema ticket sales in just two decades (Lang and Rainey, 2016). At the same time, Hollywood also faced a series of anti-trust lawsuits that dismantled the largest companies, probably leading to a restructuring of the organisations at the time (Schatz, 1999; Sedgwick and Pokorny, 2004). All or some of these events could have slowed down the cumulative evolution of film production.

In this study, we focused on 100 most popular films in each decade. This was done for both methodological and theoretical reasons. Methodology-wise, since IMDb builds up from user interest, the most popular films also have the most complete information on their crews, which was crucial for the study. Theory-wise, top films are most clearly located in the heteronomous artistic field: they most clearly compete for profit and viewers’ attention. Thus, we can expect them to be the fastest in adopting new useful techniques of filmmaking. In technology, more generally, the highest complexity is usually concentrated in a small proportion of artefacts: hammers are simple and widespread, spacecraft are complex and rare (Kelly, 2010; Hilbert, 2013). The same can be expected from films, and so the trend of increasing accumulation of innovation should be most noticeable in this part of the film industry. As the less successful usually try to learn from the more successful and the more prestigious (Jiménez and Mesoudi, 2019), we would expect less popular films to follow the lead of blockbusters in adopting new technologies, but only future empirical research can test whether this is so.

Cumulative cultural evolution has proven a useful concept for studying different domains of culture. We extended this also to arts. In the recent years we have seen an increase in large-scale quantitative studies of art, for example the attempts to measure and predict artistic success (Liu et al., 2018; Fraiberger, Sinatra, Resch, Riedl, & Barabási, 2018; Interiano et al., 2018) to detect significant trends in art history (Cutting, Brunick, DeLong, Iricinschi, & Candan, 2011; Mauch, MacCallum, Levy, & Leroi, 2015), or to uncover the internal patterns of art works (Elliott, 2016; Reagan, Mitchell, Kiley, Danforth, & Dodds, 2016). However, a major limitation of such studies is the data-driven approach they are often using. Exploratory data analysis is certainly valid as the first step in investigating rich cultural datasets, but it cannot substitute theory-driven research of the history of art. We suggest taking the next step: approaching art history with clear theoretical predictions, such as the theory of cumulative cultural evolution.

We also suggest a way of looking at art that would fit this approach. Namely, when studying long periods in art history, we can systematically analyze not just artworks themselves (this has been done in a number of studies), but also the process through which these artworks were made. The effort put into making the products by the artists has a measurable structure and is subject to change over time. For films, we found an accumulation of innovations in the structure of jobs and, most likely, the performed tasks. A similar approach could be taken when analysing the history of other creative domains, for example, comic books (where production consists of several steps, for which different people can often be responsible, e.g. penciler, inker, letterer) or popular music (e.g. songwriter, sound designer, sound mixer). This could give us a better understanding of the similarities and differences between the artistic products of different ages. A cultural evolution of art, in turn, would help us get a clearer picture of the long-term historical trajectories and our role in them.

## Supplementary material

For supplementary material accompanying this paper visit http://dx.doi.org/10.1017/ehs.2020.21. click here to view supplementary material

## Data and code availability

Data and code to reproduce the analysis and figures are available at https://osf.io/6ysda/
