# Supplementary material for: Open-ended cumulative cultural evolution of Hollywood film crews
Source: Evol Hum Sci. 2020 May 7;2:e26. doi: 10.1017/ehs.2020.21 (PMC10427440; doi:10.1017/ehs.2020.21)
Supplement: Supplementary file 1 [file S2513843X20000213sup001.pdf]

Supplementary Information Appendix

For the article "Open-ended cumulative evolution of Hollywood film crews"

Summary of the Supplementary Information Appendix

This Supplementary Information Appendix presents a detailed overview of the data collection and transformations performed, the generalized additive models (GAMs) fit, and the additional checks performed with the data. The file proceeds as follows:

- S1: Data collection. Explains how the data was collected.
- S2: Data reliability. Describes the information on data reliability included in the dataset.
- S3: Data harmonization. Explains the transformations made to dataset for the analysis.
- S4: Markers of hierarchical order. Explains how the markers of hierarchical order were detected in job titles.
- S5: Assocations between the variables. Describes the associations between the measured variables.
- S6: GAM details. Describes the GAMs reported in the paper in detail.
- S7: GAMs on markers of hierarchical order. Shows the contributions of different types of markers to the trends reported in the paper.
- S8: Checking against chance similarities. Describes an additional check made to determine whether the found accumulation of jobs could be explained by just increasing film crew sizes.
- S9: Diffusion curves in detail. Shows the diffusion curves by decade of origin.
- S10: Models of innovation space exploration
- Required libraries
- References

The data and code to reproduce the analysis and figures, both in the paper and in the supplement, are available at an Open Science Framework electronic repository here: https://osf.io/6ysda/

S1: Data collection

We collected the data on movie ratings that was used to form the sample from the downloadable datasets on the basic movie information on IMDb (retrieved April 14, 2019). The information on data completeness, used to build the sample, was given in their previous published datasets (retrieved September 9, 2017). The latter was no longer updated after November 7, 2017. The information on the film crews was manually collected for the 1,000 films in the sample from the IMDb movie website from their "Full cast and crew" listings on April 14, 2019. The markers of data completeness within the sample were also updated then for the analysis.

S2: Data reliability

Data on film crews on IMDb is marked if it is "expected to be complete" or "verified as complete". In our sample, 692 film crews had one of these markings, while 308 were not marked (see Fig. S1). 138 of the unmarked crews belonged to the 1910s and 1920s, where definite confirmation can be difficult to come by. 66 of the unmarked film crews belonged to the 2000s, where current business interests could make the data difficult to confirm. Since our sample was based on the most popular films, we expect the data to be mostly reliable even for the unmarked crews. In order to make sure that the data did not differ to a substantial degree, we applied different weights to the data points based on their expected completeness when building models (see Section S6).

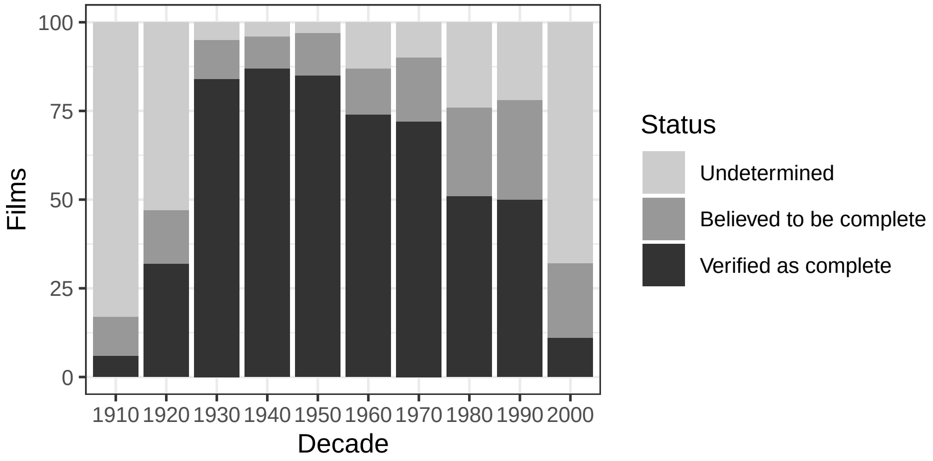

Fig. S1. The quality of data in the sample. The production crew of each film bore the mark of *Confirmed to be complete*, *Expected to be complete* or no mark. The stacked barplot shows the number of films in each category by decade.

S3: Data harmonization

Data harmonization proceeded in an iterative fashion, finding common substitutions and formatting principles behind the job titles. We removed the specifics of the jobs that were typically stored in the brackets after the job title, or following a colon (e.g., "line producer: Hong Kong", or "hair stylist: Mr. De Niro", "special makeup effects crew: Cantina sequence"). This text was used to assess whether the job should be associated with the initial release, and was removed for the analysis.

A few frequently occurring spelling variants of the same job were manually harmonized. E.g., "r&d", "R&D", "r&d;", "research & development", "research&development"; "make-up", "make up", "makeup"; "roto", "rotoscope", "rotoscoping", "roto-scope" were all replaced within the job title with a single common denominator: in these cases, "r_d", "make-up", and "roto". This was done on the basis of a manual comparison of the 11,350 unique jobs in the dataset that remained after the removal of the additional information, with a focus on more prevalent jobs.

Some of the entries contained more than one job on one line. In this case, they were split into several jobs. When a job title contained a slash separated by spaces or "and/or" (e.g., "helicopter pilot and/or camera operator"), it was split into several jobs. When a job title contained an "and", "&", or a slash separating two substrings (e.g., "painter/decorator gang boss", "giggles/howls/marmots", "hair & wig adviser; except with r&d") the parts of the job were conjoined to create several composite jobs (resulting in, e.g., "painter gang boss / decorator gang boss", "giggles / howls / marmots", "hair adviser / wig adviser"). These were then split into separate jobs for the analyses. The precise operationalization is available in the code shared with the paper.

S4: Hierarchical order of jobs

The hierarchical order of the jobs was assessed by keywords that were associated with particular roles in the hierarchy. The following parts of string were looked for within the job titles.

Superordinate positions:

"senior", "boss", "lead", "directing", "chief", "key", "executive", "supervisor", "supervising", "head", "coordinator", "co-ordinator", "foreman", "foreperson", "in charge", "manager"

Associate positions:

"contribut", "collaborat", "administrator", "associate", "advisor", "consultant", "co-" (excluding "co-ordinator")

Subordinate positions:

"assistant", "first", "second", "1st", "2nd", "additional", "technician", "junior", "intern", "secondary", "runner"

In principle, a job could have several different markings: e.g., "executive (super.) assistant (sub.) to directors", or "second (sub.) unit coordinator (super.)". As the marker "co-" was removed during data harmonization (e.g., to make sure films with two co-directors did have a director), the markers of hierarchical order were checked before data harmonization. If the job had none of the markers of hierarchical order, the job was considered unmarked.

S5: Associations between the variables

Due to the shared basis in historical trends, the measured variables are highly intercorrelated in films (see Fig. S2, top right, above the self-correlation diagonal). In order to assess their relative independence within each year, we fit a mixed effects linear model on each of the predictors with the year of release as a random effect and measured the amount of variation explained by the other variable in each predictor pair (Fig. S2, bottom left, below the self-correlation diagonal).

We find that the number of people, the number of distinct jobs, and the number of total jobs have an association $> .95$ even when controlling for year. While these measure highly overlap, we considered them separately in the analysis for conceptual clarity. We believe that the number of people or jobs should be intuitively more understandable than an aggregate value that would combine all three measures. Job title length also has a mild positive association with them, indicating a trend for films with more jobs to also have, on average, somewhat longer titles. Note that the linear relationship between the log-transformed variables also reflects the Heaps' law known from information retrieval that holds for various counts of types distributed within collections (Heaps 1978). Following this distribution, rare variants will be difficult to catch even in large samples of the collection, while common variants would be found already within small samples, indicating that at least for the core jobs the coverage will be good.

The proportion of repeated components does have a strong correlation with the number of people and jobs associated with the film (variation explained ~.63), with larger film crews offering more chance for repetitive elements to be used, while the correlation is lower for the number of unique jobs. There is a strong positive association between the repeated jobs and repeated job components as they are intrinsically linked: repetition of whole jobs will necessarily repeat their job components. However, the proportion of repeated jobs is not associated with other film crew measures (variation explained $< .20$) indicating that the repetition of elements is not a direct result of the increased crew size. For other variables, there is a also moderate association between the proportion of jobs with no hierarchical markers and the job title length (variation explained .35), while other predicted variables are fairly independent from each other (variation explained $< .20$). The first association would mean that films with more hierarchical markers had a mild tendency towards longer job titles on average. Generally, the variables are independent enough from each other to merit independent investigation.

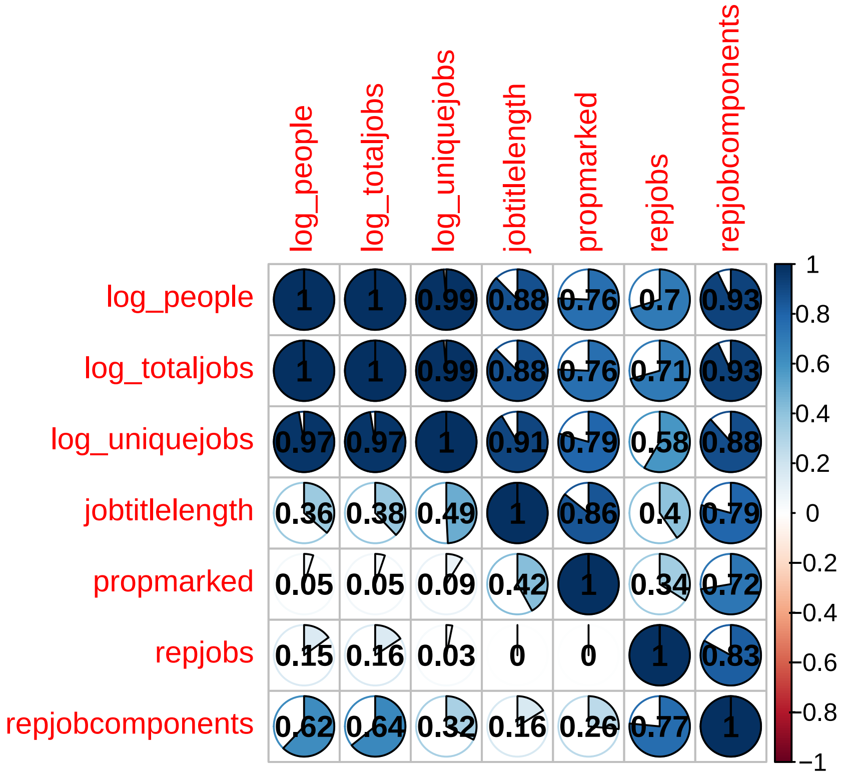

Fig. S2. Associations between the variables. Top right, above the self-correlation diagonal are raw correlations. Bottom left, below the self-correlation diagonal are the variation explained when controlling for the year of release.

S6: GAM details

We fit a Generalized Additive Model (GAM) to each of the measured variables to estimate their trends as a smooth function of their year of production. We used an adaptive smooth with the restricted maximum likelihood (REML) on smooth as a random effect to estimate a smooth that would be not overfitting or underfitting the data. We tried several basis dimension setups and in the final model we used 15 basis dimensions across all models and 5 smoothing parameters for the adaptive smooth. The results provided a good fit with the data and fulfilled the model assumptions.

In order to incorporate the information on data reliability, we varied the model weights based on the degree of confidence in the data points. We used three different parameter sets for this. In the naive model, we weighed all data points equally. In the balanced model, we gave 10% less weight to non-confirmed data points and 25% less weight to data points with no information on accuracy. In the conservative model, we gave 50% less weight to non-confirmed data points and excluded the data points with no information on accuracy.

The exact weights are given in Supplement Table S1 below.

Table S1. GAM weight parameters used

Weights on data points (DP) in GAM	DP confirmed to be accurate (n=479)	DP expected to be accurate (n=213)	No information on DP status (n=308)
Naive model	1	1	1
Conservative model	1	0.5	0
Balanced model	1	0.9	0.75

Altogether, 7 variables were modelled for the patterns of growth over time. An overview is given in Table S2.

Table S2. Overview of the model parameters

Film crew aspect	Variable measured	Transformation applied	Expected distribution
size	number of people	log	gaussian
size	number of jobs	log	gaussian
size	number of unique jobs	log	gaussian
structure	job title length	-	gaussian
structure	proportion of hierarchical jobs	-	beta
structure	reuse of whole jobs	-	beta
structure	reuse of job title components	-	beta

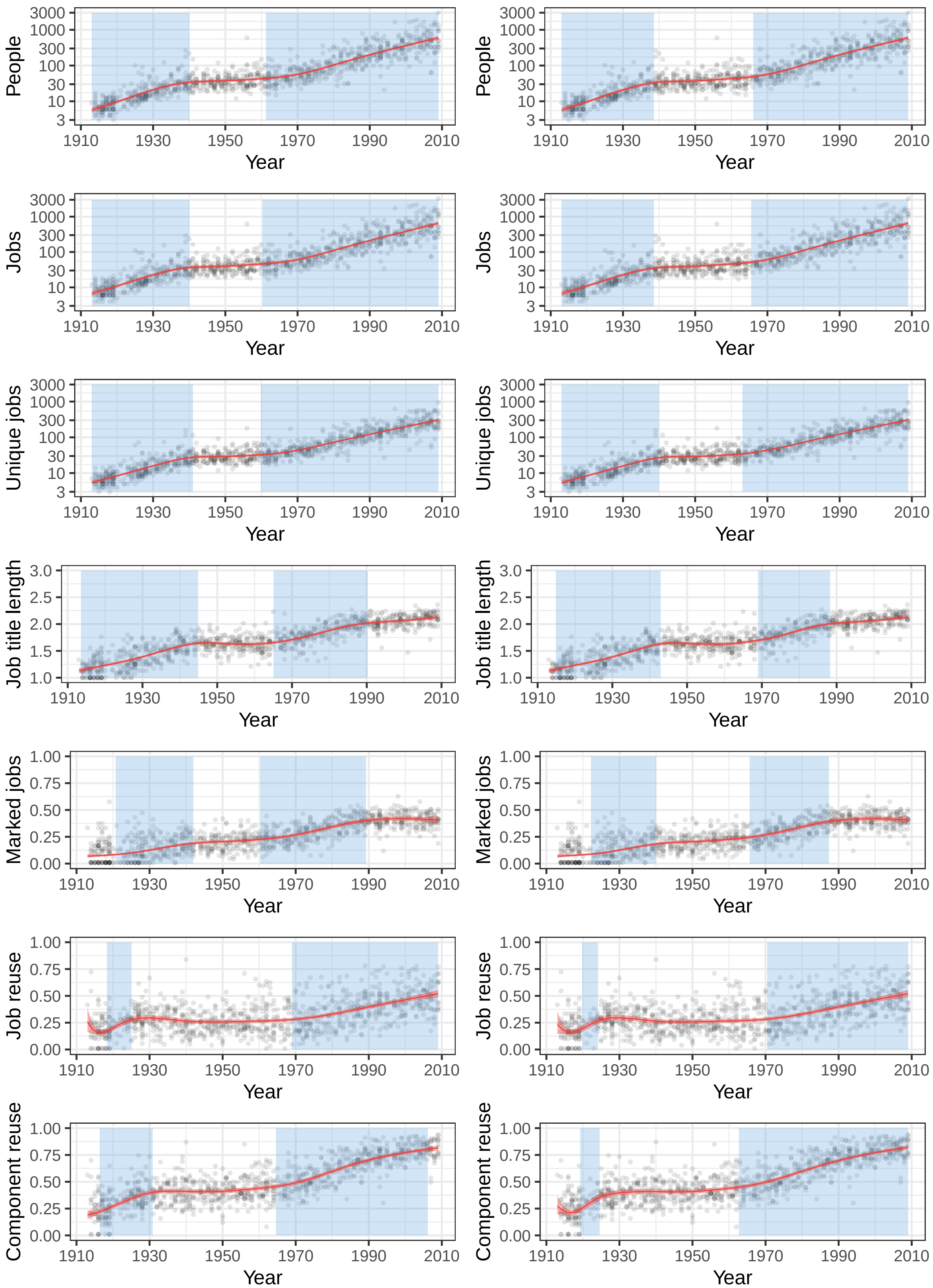

Fig. S3. Model results with $k = 15$ (left) and $k = 30$ (right). The blue shaded areas on the left are periods when the first derivation was significantly different from 0, with 95% confidence interval.

Checking model assumptions

The model checks are given for each model in Table S3. In building the models, we iteratively increased the number of basis functions (k) to get a relatively stable effective degrees of freedom and a high enough difference between it and k. In our sampling we optimized the data for a coarse view of 100 years in 10 decades, however with approximately 10 films per year (SD=4.3) it seems reasonable to include year as a continuous predictor in the model. We found 15 basis functions to provide a good model for the data. A higher k, e.g., $k = 30$, complicated the smooth function slightly (e.g., $edf = 6.33 \rightarrow 6.91$ when increasing $k = 15 \rightarrow 30$ for the number of people in the crew), however the predictions in the mean were not easily distinguishable from $k = 15$ (see Fig. S3). Since a larger k noticeably increased the credible intervals due to a larger number of possible curves included without much changes to the mean estimate, we report the models with $k = 15$ in the paper. The basis function $k = 30$ provides very similar results with slightly shorter periods of significant growth due to wider credible intervals.

The balanced and naive models give similar curves with similar effective degrees of freedom, while the conservative models provide slightly less wiggly trends due to the exclusion of many data points in the first two decades and in the last decade. The diagnostic plots for model assumptions are given in separate files in the electronic repository under figures/model.checks/: *gam_checks_bal.pdf* (balanced models), *gam_checks_con.pdf* (conservative models), *gam_checks_nai.pdf* (naive models). The models provide a reasonably good fit to the data.

Table S3. GAM checks by model

Model				Basis dimension checks		
weights	response	n	k'	edf	k-index	p-value
balanced	log(people)	1000	14	6.33	0.98	0.21
	log(jobs)	1000	14	6.09	0.98	0.32
	log(unique jobs)	1000	14	6.12	1.00	0.5
	job title length	1000	14	7.71	0.98	0.29
	hierarchical jobs	1000	14	6.28	1.01	0.6
	repeated jobs	1000	14	6.42	1.01	0.61
	repeated job title components	1000	14	6.59	1.02	0.7
conservative	log(people)	715	14	5.42	1.00	0.58
	log(jobs)	715	14	5.13	1.00	0.47
	log(unique jobs)	715	14	5.33	1.04	0.83
	job title length	715	14	7.63	1.03	0.82
	hierarchical jobs	715	14	3.73	1.02	0.7
	repeated jobs	715	14	4.29	0.94	0.06
	repeated job title components	715	14	4.23	0.97	0.2
naive	log(people)	1000	14	6.33	0.97	0.21
	log(jobs)	1000	14	6.33	0.98	0.3
	log(unique jobs)	1000	14	6.14	0.99	0.42
	job title length	1000	14	7.66	0.98	0.24
	hierarchical jobs	1000	14	6.71	1.01	0.67
	repeated jobs	1000	14	6.64	1.00	0.52
	repeated job title components	1000	14	7.19	1.03	0.86

Model summaries

Each model showed a significant effect of the smoothed year across the period, demonstrating a pattern of growth for all response variables over the period.

The main results are given in the Table S4 below. The deviance explained was very high (>0.8) for the balanced and naive models on film crew size. Relatively high (>0.7) for job title length and repeated job title components, medium (~0.6) for the proportion of hierarchical jobs and low (~0.3) for repeated whole job titles. The deviance explained was similar, but slightly lower in the conservative models that excluded almost one third of the data points.

Table S4. GAM summaries by model

Model				s(year)			
weights	response	n	dev.expl	edf	Ref.df	F / Chi.sq	p-value
balanced	log(people)	1000	0.83	6.33	7.48	649.12	< 0.001 ***
	log(jobs)	1000	0.83	6.09	7.20	653.77	< 0.001 ***
	log(unique jobs)	1000	0.86	6.12	7.21	841.22	< 0.001 ***
	job title length	1000	0.78	7.71	8.99	390.24	< 0.001 ***
	hierarchical jobs	1000	0.65	6.28	7.50	1265.68	< 0.001 ***
	repeated jobs	1000	0.32	6.42	7.50	382.31	< 0.001 ***
	repeated job title components	1000	0.70	6.59	7.81	1626.83	< 0.001 ***
conservative	log(people)	715	0.75	5.42	6.49	333.43	< 0.001 ***
	log(jobs)	715	0.74	5.13	6.15	334.55	< 0.001 ***
	log(unique jobs)	715	0.79	5.33	6.36	418.47	< 0.001 ***
	job title length	715	0.67	7.63	8.95	158.82	< 0.001 ***
	hierarchical jobs	715	0.54	3.73	4.37	685.10	< 0.001 ***
	repeated jobs	715	0.21	4.29	5.22	164.74	< 0.001 ***
	repeated job title components	715	0.61	4.23	5.11	816.34	< 0.001 ***
naive	log(people)	1000	0.84	6.33	7.48	695.51	< 0.001 ***
	log(jobs)	1000	0.84	6.33	7.48	678.82	< 0.001 ***
	log(unique jobs)	1000	0.87	6.14	7.22	905.77	< 0.001 ***
	job title length	1000	0.80	7.66	8.94	429.72	< 0.001 ***
	hierarchical jobs	1000	0.66	6.71	7.97	1453.57	< 0.001 ***
	repeated jobs	1000	0.34	6.64	7.72	449.47	< 0.001 ***
	repeated job title components	1000	0.71	7.19	8.30	1880.15	< 0.001 ***

In order to find the periods of significant change, we estimated the derivatives of the fitted spline with the method of finite differences: we took the predictions of estimated means at two close time points and calculated the difference between them. We did this for 300 points across the observed 100-year period. Based on this, we simulated 200 model fits and estimated the 95% simultaneous credible interval of the smooths.

The periods of significant change are the time periods where the simultaneous credible interval on the first derivative does not include zero. These intervals were obtained by simulation from the posterior distribution of the first derivative. A 95% confidence interval here contains in its entirety 95% of all random draws from the posterior distribution.

The results of the models are provided below (Fig. S3) with the comparison of the three sets of models. On the left is the model fit along with the data and confidence intervals, with the periods of growth shaded blue. On the right are the estimated first derivatives of that plot across the time period. Whenever the first derivatives did not include 0 but were bigger than it, the significant period of growth was marked. At no point did any of the measures show a significant trend of decrease.

Comparisons

The three different ways to include information on data completeness give very similar results for almost all parameters. Due to the removal of many data points in the first two decades, the conservative models differ in their estimations of hierarchical order, job title length, job reuse and job component reuse, offering predicted means closer to the subsequent decades. For job reuse and job component reuse, the first growth period situated around 1920s would then disappear. For markers of hierarchical order, this extends the trend slightly to the beginning of the period and for job title length, this slightly shortens the trend. However, given that this is due to most data points in 1910s not having been marked for data completeness, we follow the models that do rely on all data points as better data may be difficult to come by on this and it is reasonable to expect that the data on the most popular films is still fairly reliable. For the later periods, including the 2000s, the models offer mostly the same predictions. In the paper, the results of the balanced model are reported.

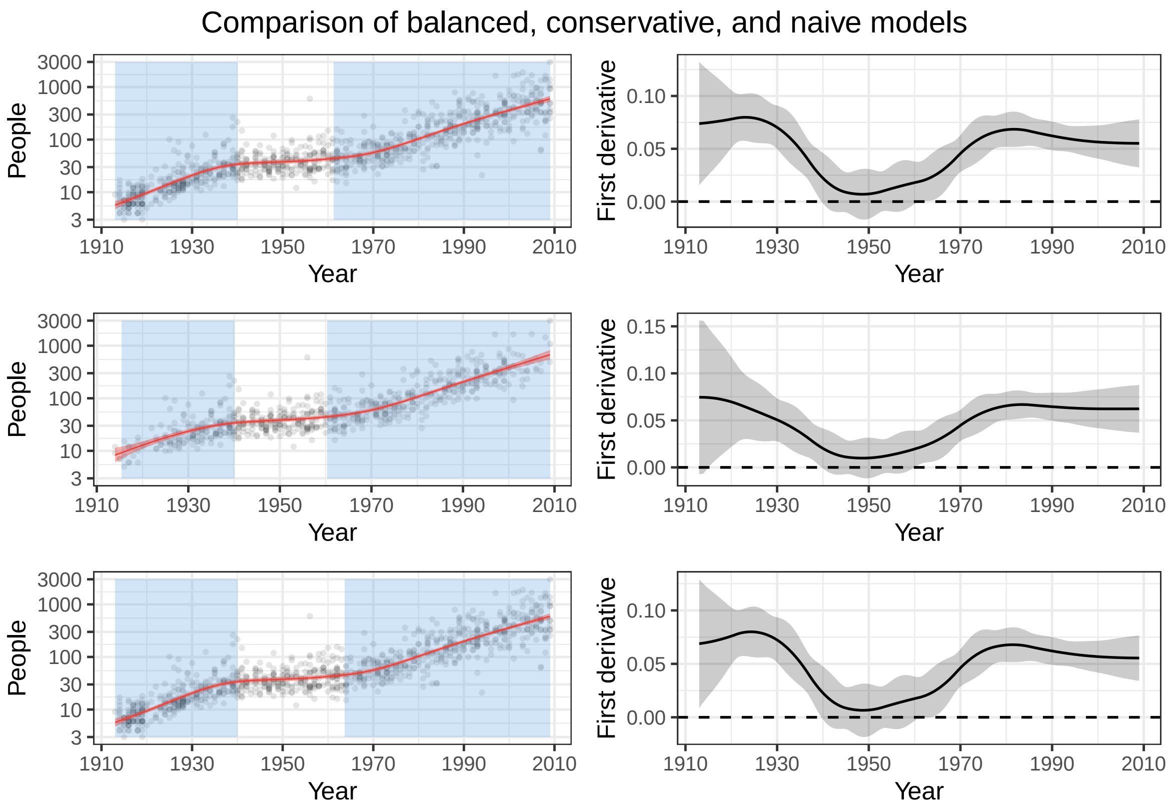

Fig. S4a. GAM results on the number of people per film (n = 1,000). The predicted values with 95% confidence interval on the left, first derivation on the right. The blue shaded areas on the left are periods when the first derivation was significantly different from 0, with 95% confidence interval. The top row - balanced; middle row - conservative; bottom row - naive models.

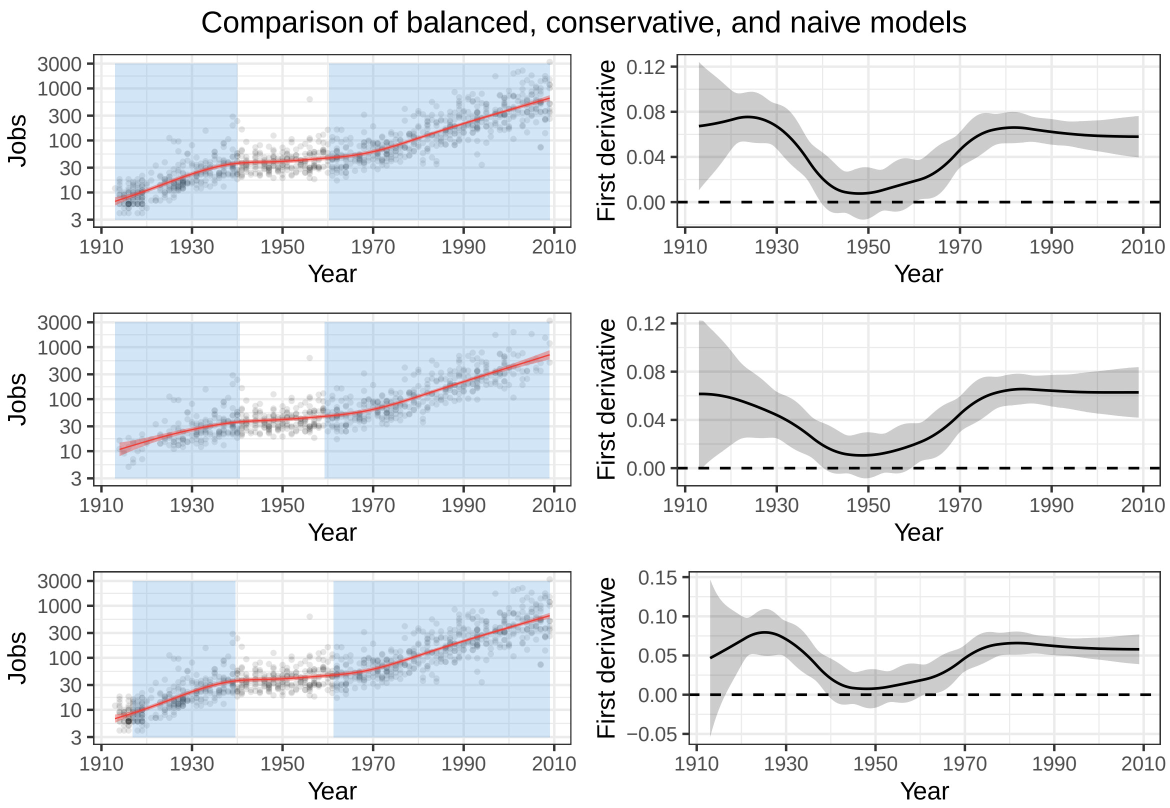

Fig. S4b. GAM results on the number of jobs per film (n = 1,000). Figure is structured as Fig. S4a.

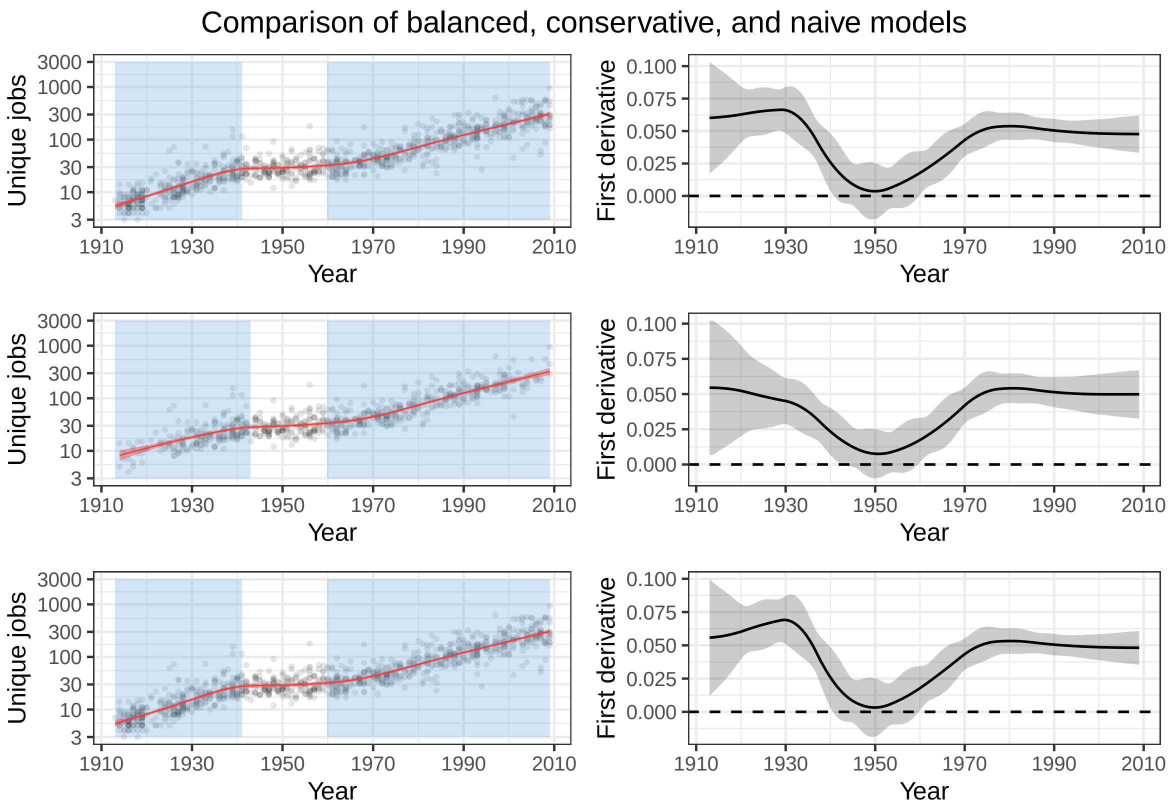

Fig. S4c. GAM results on the number of unique jobs per film (n = 1,000). Figure is structured as Fig. S4a.

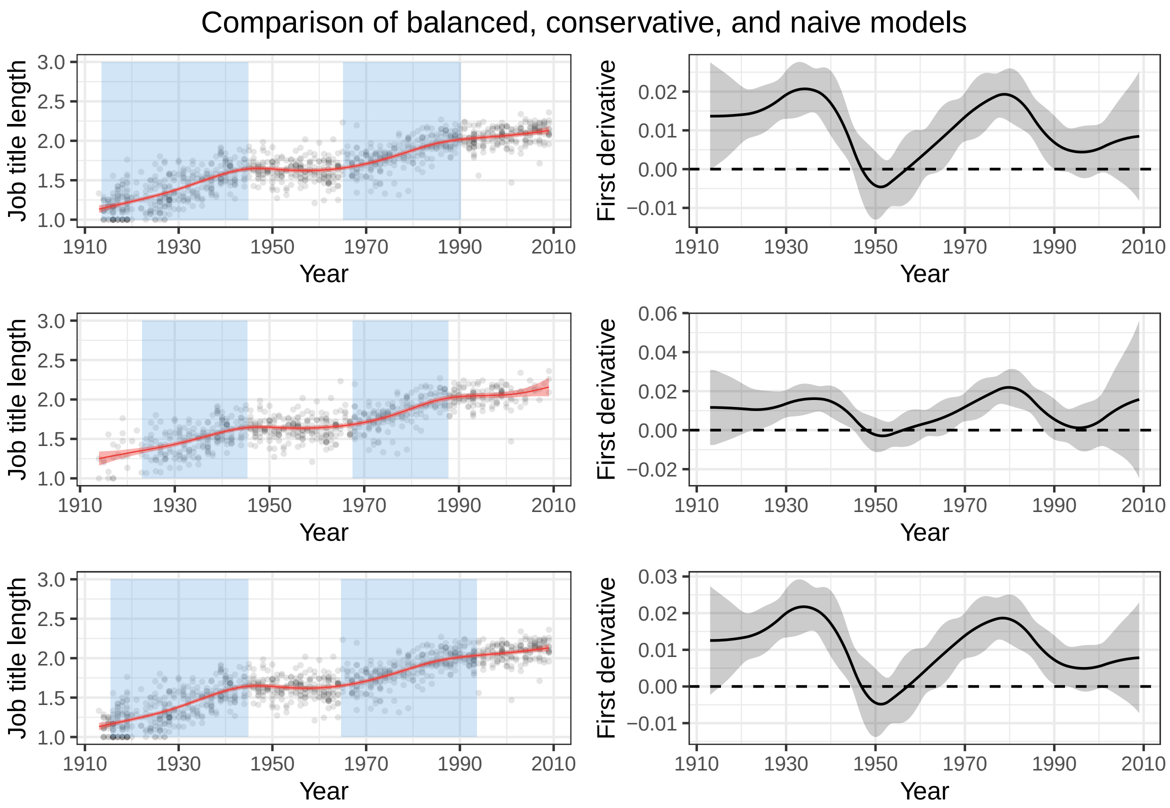

Fig. S4d. GAM results on the mean job title length per film (n = 1,000). Figure is structured as Fig. S4a.

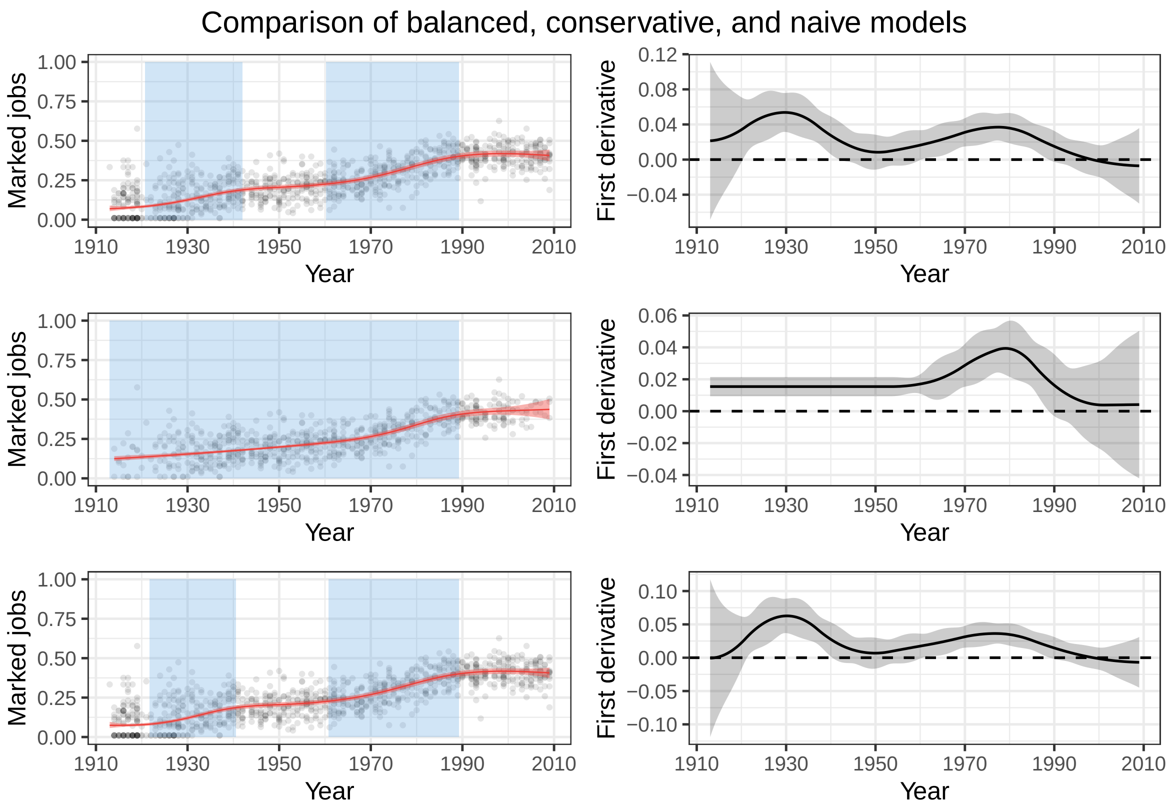

Fig. S4e. GAM results on the proportion of marked jobs per film (n = 1,000). Figure is structured as Fig. S4a.

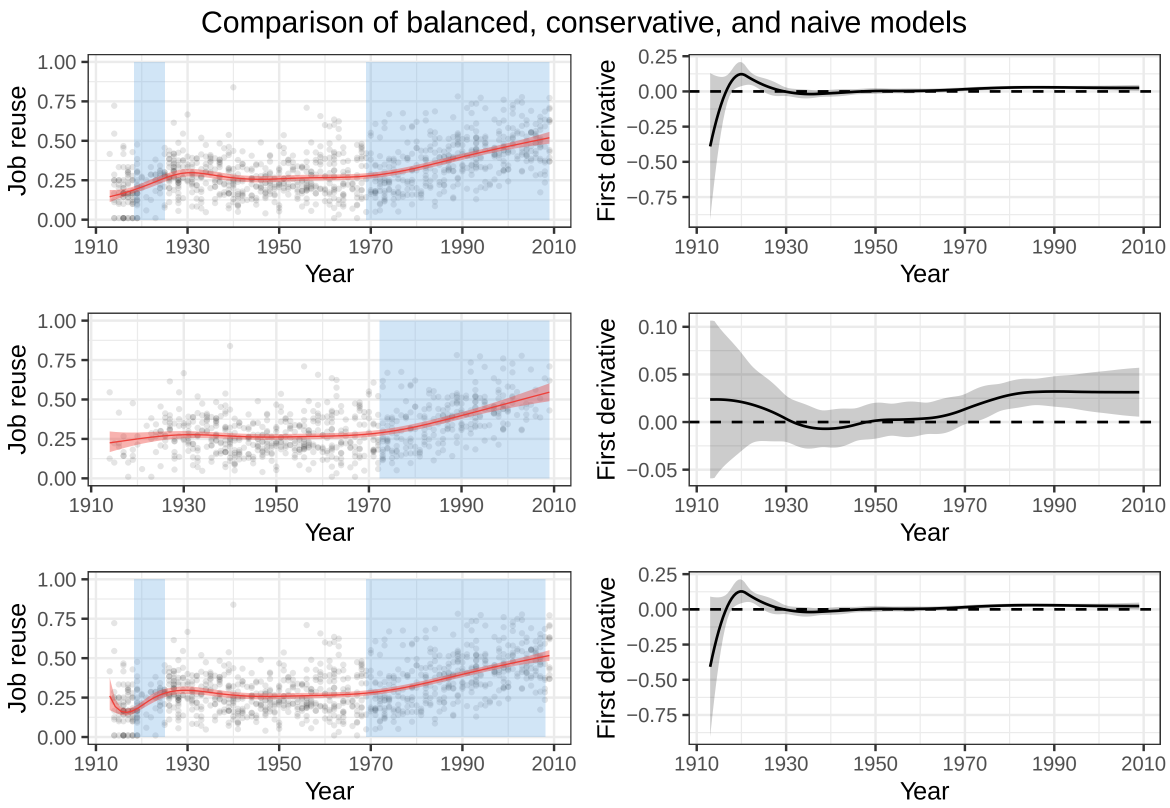

Fig. S4f. GAM results on the ratio of job reuse per film ($n = 1{,}000$). Figure is structured as Fig. S4a.

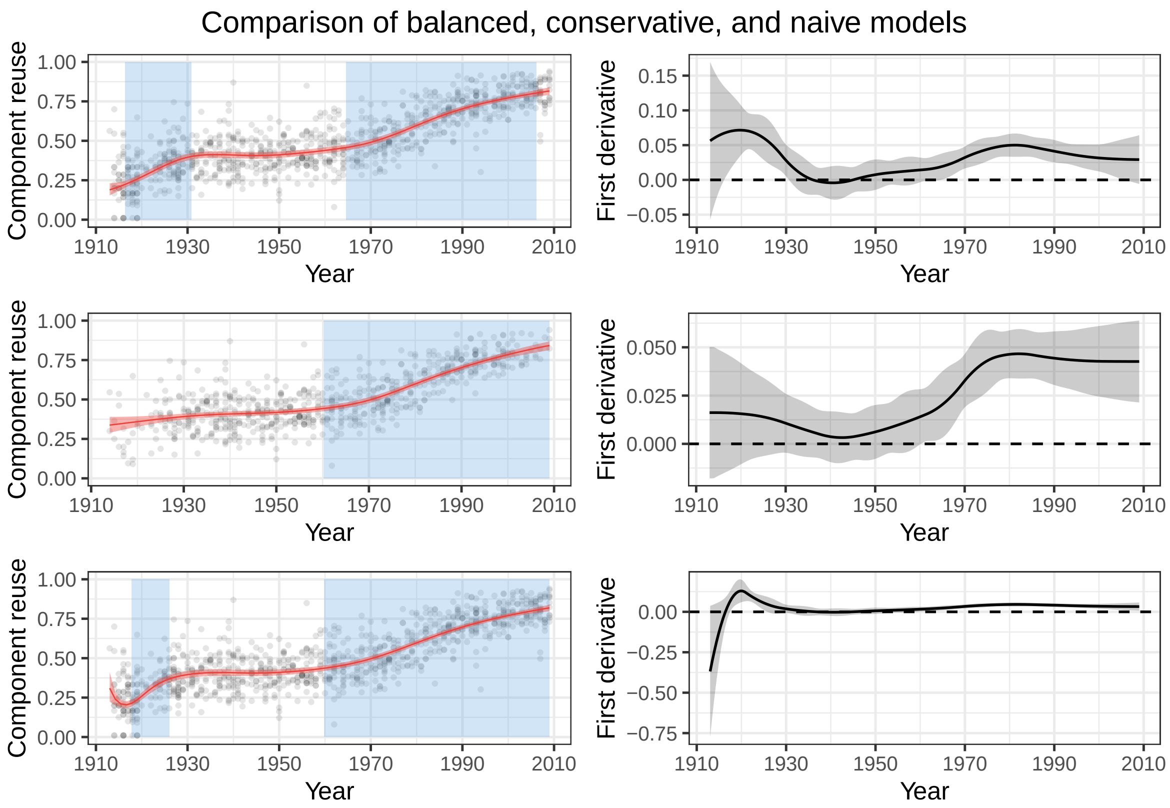

Fig. S4f. GAM results on the ratio of job title component reuse per film (n = 1,000). Figure is structured as Fig. S4a.

S7: GAMs on markers of hierarchical order

The paper reported a non-linear trend of increase in the proportion of jobs with the markers of hierarchical order. This in turn comprised of the trends of the three types of jobs: superordinate, equal, and subordinate. In order to better understand the trend, we fit a GAM on each of them with the same model parameters as the model of hierarchical jobs, with balanced weights on the data points (see Fig. S4 for results). We found that this trend was comprised of a gradual linear increase of superordinate jobs until the 2000s and the punctuated growth of subordinate jobs, with a slower increase until the 1940s and a quicker increase from 1964 to 1987. The proportion of associate jobs remained quite low throughout the period.

Checking model assumptions

The model checks are given for each model in Table S5. The number of basis dimensions was chosen as 15, same as in other models. The diagnostic plots for model assumptions are given in a separate file in the electronic repository under figures/model.checks/: *gam_checks_bal_hier.pdf.* The models provide a reasonable fit to the data.

Table S5. GAM checks by marker type

Model				Basis dimension checks		
weights	response	n	k'	edf	k-index	p-value
balanced	Superordinate jobs	1000	14	2.72	0.99	0.32
	Subordinate jobs	1000	14	5.50	0.96	0.11
	Equal jobs	1000	14	4.29	0.83	0 ***
	All jobs	1000	14	6.28	1.01	0.62

Model summaries

Each model showed a significant effect of the smooth function of the year across the period, demonstrating a pattern of growth for all response variables over the observed period (Fig. S5). The deviance explained by the model was high for superordinate jobs (.60), subordinate jobs (.52) and all the markers of hierarchical order together (.65). The proportion of associate jobs was not well predicted by the smooth of the year (deviance explained .10). The main results are given in the Table S6 below.

We estimated the derivatives in the same way as with other models. The results of the models are provided below (Fig. S5). On the left is the model fit along with the data and confidence intervals, with the periods of growth shaded blue. On the right is are the estimated first derivatives of that plot across the time period. Whenever the first derivatives did not include 0 but was bigger than it, the significant period of growth was marked. At no point did any of the measures show a significant trend of decrease.

Table S6. GAM summaries by marker type

Model				s(year)			
weights	response	n	dev.expl	edf	Ref.df	Chi.sq	p-value
balanced	Superordinate jobs	1000	0.60	2.72	3.32	1069.81	< 0.001 ***
	Subordinate jobs	1000	0.52	5.50	6.57	867.06	< 0.001 ***
	Equal jobs	1000	0.10	4.29	4.97	84.62	< 0.001 ***
	All jobs	1000	0.65	6.28	7.50	1265.68	< 0.001 ***

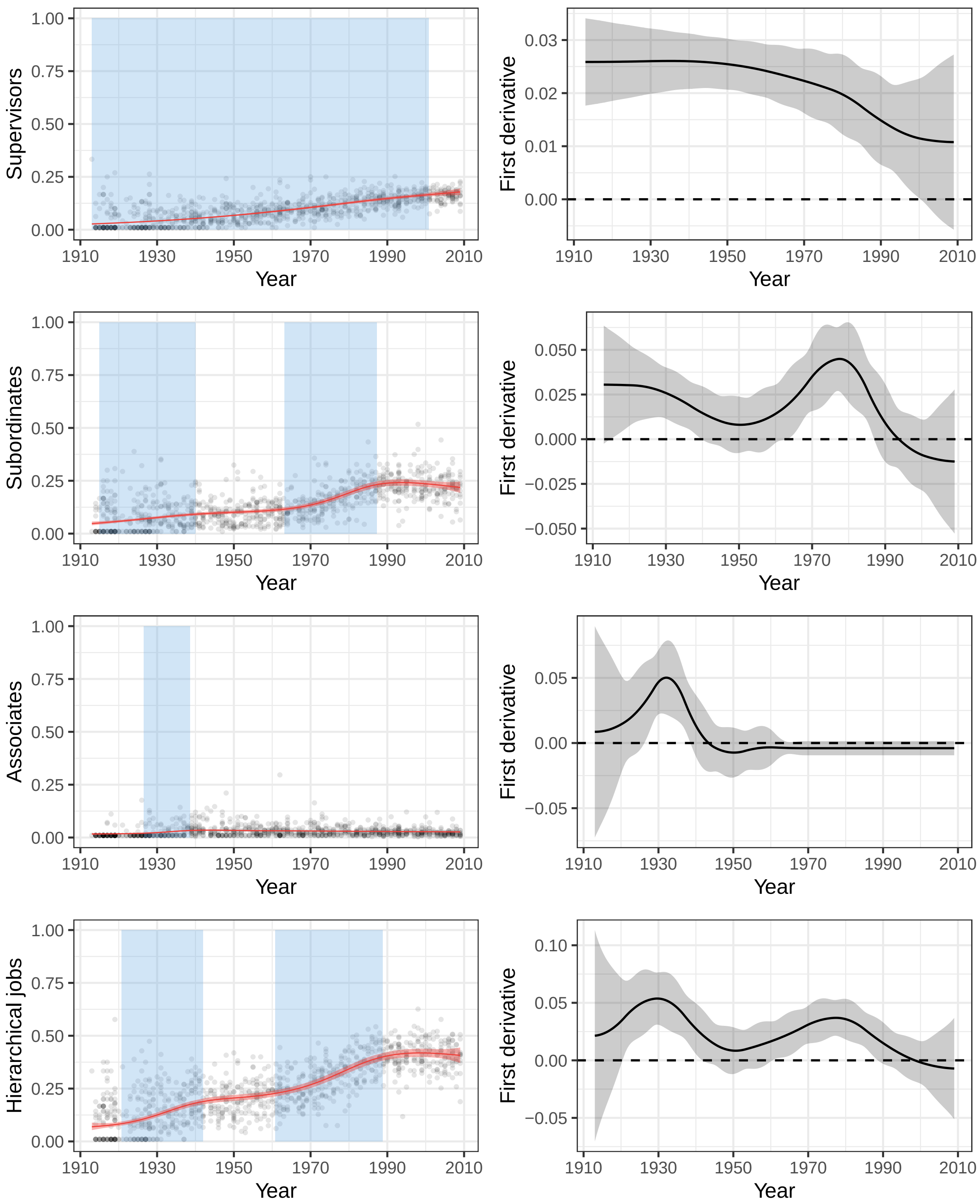

Fig. S5. GAM results on the different types of markers of hierarchical order ($n = 1{,}000$). The predicted values with 95% confidence interval on the left, first derivation on the right. The blue shaded areas on the left are periods when the first derivation was significantly different from 0, with 95% confidence interval. From top to bottom the measures depicted are 1) Jobs with superordinate markers; 2) Jobs with subordinate markers; 3) Jobs with neutral markers; 4) Unmarked jobs over time.

S8: Checking against chance similarities

In order to check that the growth of the number of central jobs is not simply due to bigger film crews increasing the number of chance similarities, we created 1,000 random permutations of the dataset where each film was given a random subset of unique jobs present in that decade that matched the number of unique jobs in that film. In both our sample and the random datasets the distribution of jobs is power-law-like: most jobs are relatively rare between films, while few jobs are present in many films. Always, less than 15% of the jobs are present in more than 15% of films. Fig. S6a plots the cumulative distributions of jobs of our data and one random sample across the decades. The slope of the distribution is much steeper for the generated dataset. Randomly, there are very few jobs in more than 15% of films per decade, while in our data there are more than 5% of jobs in at least 15% of films for all decades. Over time, the distribution becomes flatter and smoother as more unique jobs are added in both datasets, e.g., in the 1910s, 5% of jobs were in 30% or more films, while in the 2000s, only 2.5% of them were in 30% or more films in our sample.

Fig. S6b plots the mean number of jobs in more than 10% of the films in each decade for all 1,000 generated datasets along with the data from our sample. Due to the number of unique jobs in each decade increasing along with the film crew sizes, the number of jobs shared by chance between many films stays roughly the same throughout the period, which, except for the first decade, is decisively lower than in our sample ($M_{\mathrm{MEAN}} = 12.7$, $SE_{\mathrm{MEAN}} = 6.1$, $M_{\mathrm{SD}} = 3.0$, $SE_{\mathrm{SD}} = 0.7$ between decades). Across the samples, there were only a few occasions of jobs shared by more than 20% of films, and it was never more than one job, always in the 1910s. This indicates that the growth of the number of jobs shared between films is indeed due to an accumulation of innovations that become preferentially used between films.

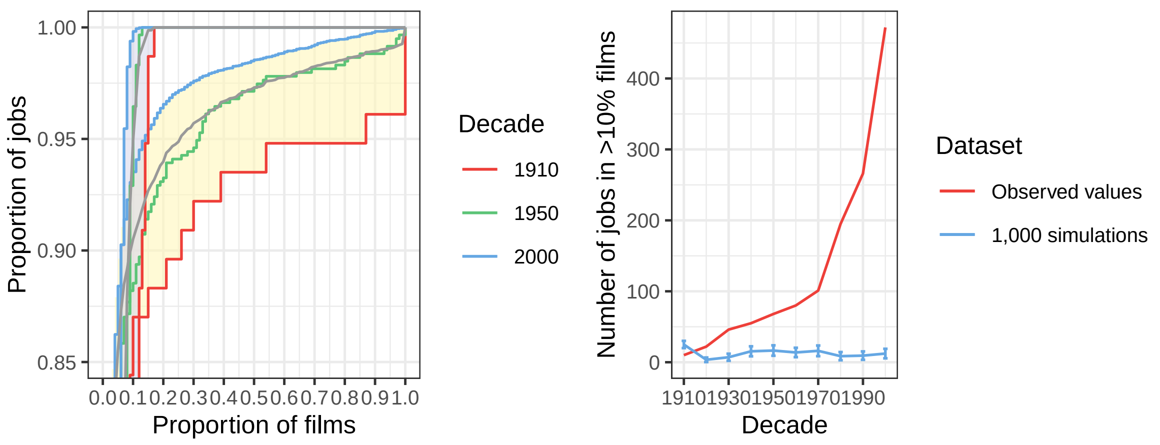

Fig. S6. Results of randomization. a) The cumulative distribution of jobs between films. The dataset studied is shown with yellow background and one generated dataset is shown with purple background. Grey lines show the mean across decades, selected decades are shown in a distinct lines. b) The number of jobs shared by more than 10% of films in the decade. The red line shows our sample across decades, the blue line shows the generated 1,000 datasets with error bars at ± 2SD from the mean.

S9: Diffusion curves in detail

Fig. S7 shows the adoption curves of the jobs that were in at least 20% films in the 2000s, separately for each decade of first occurrence. The figure shows that apart from a few jobs from the very first decade that reached very high popularity right away, the growth in popularity for most of the jobs was gradual.

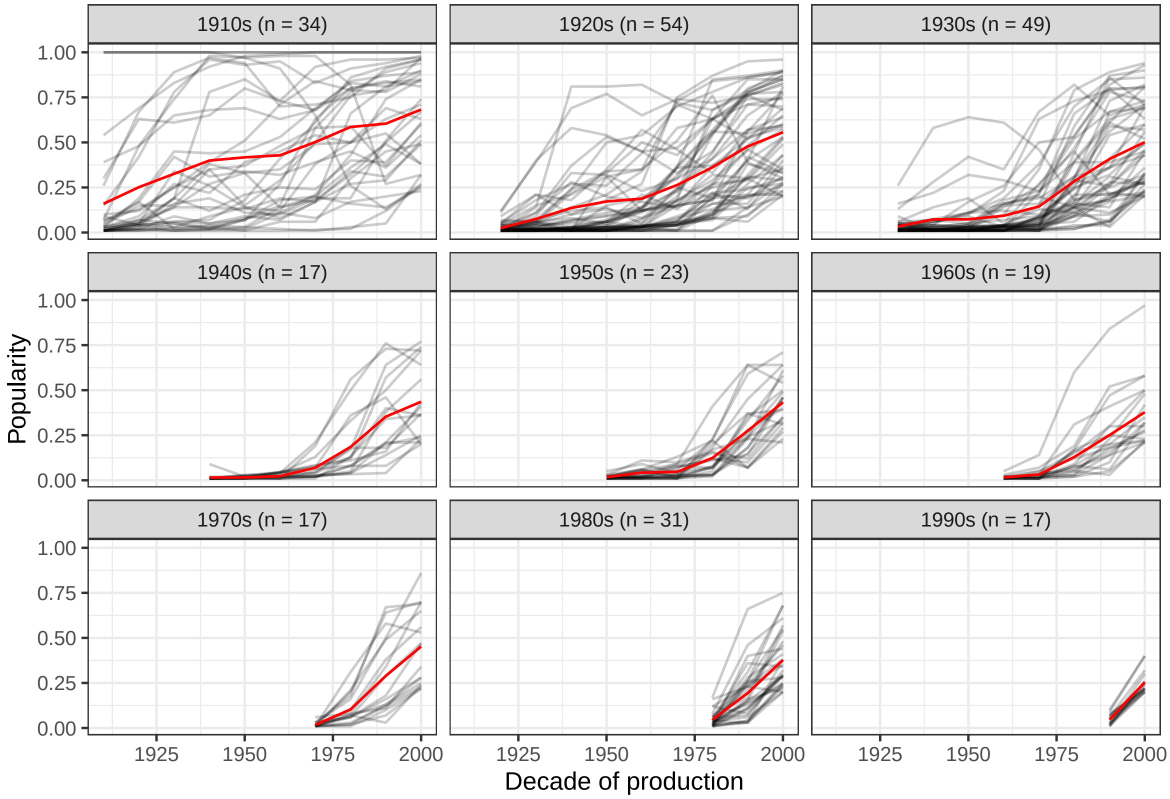

Fig. S7. Diffusion curves separated by decade of first occurrence from Fig. 3a. Red line shows the mean of the decade, grey lines trace the diffusion of each particular job across decades.

S10: Models of innovation space exploration

To test the association of the number of inventions with the variety originating from earlier generations we counted all the distinct jobs per decade and divided them into two parts: ones invented in the same decade and ones reused from earlier decades. We log-transformed these values to reduce the influence of larger values and fit a linear regression model to estimate the association between the two. Through model criticism and selection, we added the total growth rate compared to the previous decade as an additional predictor, which significantly improved the model. See the code attached in the SI Appendix for details. The model provided a good fit to the data explained 96% of the variation in the data ($\text{beta}_{\text{variety}} = 0.87$ 95% CI [0.71 – 1.04], $\text{beta}_{\text{growth}} = 0.97$ 95% CI [0.54 – 1.39], $F\ (2,6) = 103.9$, $p < 0.01$, $R^2 = 0.96$). The final formula was the following.

log_new_jobs ~ log_old_jobs + perc_increase

The model diagnostic plots are given in a separate file figures/model.checks/: *lm_checks.pdf.*

To test the same association within thematic groups and to estimate the influence of local variation on the generation of innovations, we grouped the data into thematic clusters based on shared words within the job title and constructed a mixed effects model to determine the relationship within thematic clusters. For this analysis, we only included the clusters that had at least 10 jobs within a cluster, meaning that at least some exploration had been done in that innovation space. There were altogether 352 such thematic clusters (distinct jobs in cluster median = 28, IQR = 16-56, range = 10-1266). Based on model criticism and selection, we found the best fit in adding both the random intercept and slope for each group to the ordinary least squares regression model specified above. We found the association to be strong ($\text{beta}_{\text{variety}} = 0.77$ 95% CI [0.72 – 0.82], $\text{beta}_{\text{growth}} = 0.71$ 95% CI [0.62 – 0.80], with marginal $R^2 = 0.57$ and conditional $R^2 = 0.70$), demonstrating the close link between the variation already present in the population with inventions produced in this area of culture. This eventually gave the following formula.

log_new_jobs ~ log_old_jobs + perc_increase + (1 + log_old_jobs | , job_component)

The model diagnostic plots and overview are given in a separate file figures/model.checks/: *lmer_checks.pdf.*

Required libraries

- Base R R version 3.6.3 (2020-02-29) (R Core Team 2019; Allaire et al. 2019)
- data.table 1.12.6 (Dowle & Srinivasan 2019)
- ggplot2 3.2.1 (Wickham 2016)
- dplyr 0.8.4 (Wickham et al. 2019)
- zoo 1.8.6 (Zeileis & Grothendieck 2005)
- mgcv 1.8.31 (Wood 2011; Wood et al. 2016; Wood 2004; Wood 2017)
- mgcViz0.1.4 (Fasiolo et al. 2018)
- lme4 1.1.21 (Bates et al. 2015)
- corrplot 0.84 (Wei & Simko 2017)
- MuMIn 1.43.15 (Bartoń 2018)
- kableExtra 1.1.0 (Zhu 2019)
- scales 1.1.0 (Wickham 2018)
- stringr 1.4.0 (Wickham 2019a)
- gratia 0.2.8 (Simpson 2019)
- colorspace 1.4.1 (Zeileis et al. 2019; Zeileis, Hornik & Murrell 2009; Stauffer et al. 2009)
- RColorBrewer 1.1.2 (Neuwirth 2014)
- igpraph 1.2.4.2 (Csardi & Nepusz 2006)
- ggraph 2.0.0 (Pedersen 2018)
- tidygraph 1.1.2 (Pedersen 2019)
- ggrepel 0.8.1 (Slowikowski 2018)
- RcmdrPlugin.KMggplot2 0.2.6 (Sou & Nagashima 2018)
- forcats 0.4.0 (Wickham 2019b)
- cowplot 1.0.0 (Wilke 2019)
- sjPlot 2.8.2 (Lüdecke 2020)
- sjmisc 2.8.3 (Lüdecke 2018)
- lme4 1.1.21 (Bates et al. 2015)
- olsrr 0.5.2 (Hebbali 2018)

References

Allaire, JJ, Yihui Xie, Jonathan McPherson, Javier Luraschi, Kevin Ushey, Aron Atkins, Hadley Wickham, Joe Cheng & Winston Chang. 2019. *Rmarkdown: Dynamic documents for r.* https://CRAN.R-project.org/package=rmarkdown.

Bartoń, Kamil. 2018. *MuMIn: Multi-model inference.* https://CRAN.R-project.org/package=MuMIn.

Bates, Douglas, Martin Mächler, Ben Bolker & Steve Walker. 2015. Fitting linear mixed-effects models using lme4. *Journal of Statistical Software* 67(1). 1–48. https://doi.org10.18637/jss.v067.i01.

Csardi, Gabor & Tamas Nepusz. 2006. The igraph software package for complex network research. *InterJournal* Complex Systems. 1695. http://igraph.org.

Dowle, Matt & Arun Srinivasan. 2019. *Data.table: Extension of 'data.frame'.* https://CRAN.R-project.org/package=data.table.

Fasiolo, Matteo, Raphael Nedellec, Yannig Goude & Simon N. Wood. 2018. Scalable visualisation methods for modern generalized additive models. *Arxiv preprint.* https://arxiv.org/abs/1707.03307.

Heaps, H. S. 1978. *Information retrieval: Computational and theoretical aspects.* USA: Academic Press, Inc.

Hebbali, Aravind. 2018. *Olsrr: Tools for building ols regression models.* https://CRAN.R-project.org/package=olsrr.

Lüdecke, Daniel. 2018. Sjmisc: Data and variable transformation functions. *Journal of Open Source Software* 3(26). 754. https://doi.org10.21105/joss.00754.

Lüdecke, Daniel. 2020. *SjPlot: Data visualization for statistics in social science.* https://doi.org10.5281/zenodo.1308157. https://CRAN.R-project.org/package=sjPlot.

Neuwirth, Erich. 2014. *RColorBrewer: ColorBrewer palettes.* https://CRAN.R-project.org/package=RColorBrewer.

Pedersen, Thomas Lin. 2018. *Ggraph: An implementation of grammar of graphics for graphs and networks.* https://CRAN.R-project.org/package=ggraph.

Pedersen, Thomas Lin. 2019. *Tidygraph: A tidy api for graph manipulation.* https://CRAN.R-project.org/package=tidygraph.

R Core Team. 2019. *R: A language and environment for statistical computing.* Vienna, Austria: R Foundation for Statistical Computing. https://www.R-project.org/.

Simpson, Gavin L. 2019. *Gratia: Graceful 'ggplot'-based graphics and other functions for gams fitted using 'mgcv'.* https://gavinsimpson.github.io/gratia.

Slowikowski, Kamil. 2018. *Ggrepel: Automatically position non-overlapping text labels with 'ggplot2'.* https://CRAN.R-project.org/package=ggrepel.

Sou, Triad & Kengo Nagashima. 2018. *RcmdrPlugin.KMggplot2: R commander plug-in for data visualization with 'ggplot2'.* https://CRAN.R-project.org/package=RcmdrPlugin.KMggplot2.

Stauffer, Reto, Georg J. Mayr, Markus Dabernig & Achim Zeileis. 2009. Somewhere over the rainbow: How to make effective use of colors in meteorological visualizations. *Bulletin of the American Meteorological Society* 96(2). 203–216. https://doi.org10.1175/BAMS-D-13-00155.1.

Wei, Taiyun & Viliam Simko. 2017. *R package "corrplot": Visualization of a correlation matrix.* https://github.com/taiyun/corrplot.

Wickham, Hadley. 2016. *Ggplot2: Elegant graphics for data analysis.* Springer-Verlag New York. http://ggplot2.org.

Wickham, Hadley. 2018. *Scales: Scale functions for visualization.* https://CRAN.R-project.org/package=scales.

Wickham, Hadley. 2019a. *Stringr: Simple, consistent wrappers for common string operations.* https://CRAN.R-project.org/package=stringr.

Wickham, Hadley. 2019b. *Forcats: Tools for working with categorical variables (factors).* https://CRAN.R-project.org/package=forcats.

Wickham, Hadley, Romain François, Lionel Henry & Kirill Müller. 2019. *Dplyr: A grammar of data manipulation.* https://CRAN.R-project.org/package=dplyr.

Wilke, Claus O. 2019. *Cowplot: Streamlined plot theme and plot annotations for 'ggplot2'.* https://CRAN.R-project.org/package=cowplot.

Wood, S. N. 2004. Stable and efficient multiple smoothing parameter estimation for generalized additive models. *Journal of the American Statistical Association* 99(467). 673–686.

Wood, S. N. 2011. Fast stable restricted maximum likelihood and marginal likelihood estimation of semiparametric generalized linear models. *Journal of the Royal Statistical Society (B)* 73(1). 3–36.

Wood, S.N. 2017. *Generalized additive models: An introduction with r.* 2nd edn. Chapman; Hall/CRC.

Wood, S.N., N., Pya & B. S"afken. 2016. Smoothing parameter and model selection for general smooth models (with discussion). *Journal of the American Statistical Association* 111. 1548–1575.

Zeileis, Achim, Jason C. Fisher, Kurt Hornik, Ross Ihaka, Claire D. McWhite, Paul Murrell, Reto Stauffer & Claus O. Wilke. 2019. *colorspace: A toolbox for manipulating and assessing colors and palettes.* ArXiv. arXiv.org E-Print Archive. http://arxiv.org/abs/1903.06490.

Zeileis, Achim & Gabor Grothendieck. 2005. Zoo: S3 infrastructure for regular and irregular time series. *Journal of Statistical Software* 14(6). 1–27. https://doi.org10.18637/jss.v014.i06.

Zeileis, Achim, Kurt Hornik & Paul Murrell. 2009. Escaping RGBland: Selecting colors for statistical graphics. *Computational Statistics & Data Analysis* 53(9). 3259–3270. https://doi.org10.1016/j.csda.2008.11.033.

Zhu, Hao. 2019. *KableExtra: Construct complex table with 'kable' and pipe syntax*. https://CRAN.R-project.org/package=kableExtra.
